# Nonpharmaceutical Measures for Pandemic Influenza in Nonhealthcare Settings—Social Distancing Measures

**DOI:** 10.3201/eid2605.190995

**Published:** 2020-05

**Authors:** Min W. Fong, Huizhi Gao, Jessica Y. Wong, Jingyi Xiao, Eunice Y.C. Shiu, Sukhyun Ryu, Benjamin J. Cowling

**Affiliations:** University of Hong Kong, Hong Kong, China

**Keywords:** influenza, pandemic influenza, influenza viruses, viruses, pandemic, respiratory infections, nonpharmaceutical measures, nonhealthcare settings, social distancing measures, public health

## Abstract

Influenza virus infections are believed to spread mostly by close contact in the community. Social distancing measures are essential components of the public health response to influenza pandemics. The objective of these mitigation measures is to reduce transmission, thereby delaying the epidemic peak, reducing the size of the epidemic peak, and spreading cases over a longer time to relieve pressure on the healthcare system. We conducted systematic reviews of the evidence base for effectiveness of multiple mitigation measures: isolating ill persons, contact tracing, quarantining exposed persons, school closures, workplace measures/closures, and avoiding crowding. Evidence supporting the effectiveness of these measures was obtained largely from observational studies and simulation studies. Voluntary isolation at home might be a more feasible social distancing measure, and pandemic plans should consider how to facilitate this measure. More drastic social distancing measures might be reserved for severe pandemics.

Experiences from previous influenza pandemics, in particular the 2009–10 pandemic, have demonstrated that we cannot expect to contain geographically the next influenza pandemic in the location it emerges, nor can we expect to prevent international spread of infection for more than a short period. Vaccines are not expected to be available during the early stage of the next pandemic (*1*), and stockpiles of antiviral drugs will be limited, mostly reserved for treating more severe illnesses and for patients at higher risk for influenza complications. Therefore, nonpharmaceutical interventions (NPIs), such as social distancing (*2*), will be heavily relied on by health authorities to slow influenza transmission in the community, with 3 desired outcomes (Figure). The first outcome would be to delay the timing of the peak of infections to buy time for preparations in the healthcare system, the second to reduce the size of the epidemic peak so that the healthcare system is not overwhelmed, and the third to spread infections over a longer time period, enabling better management of those cases and the potential for vaccines to be used at least later in the epidemic to reduce impact.

**Figure:**
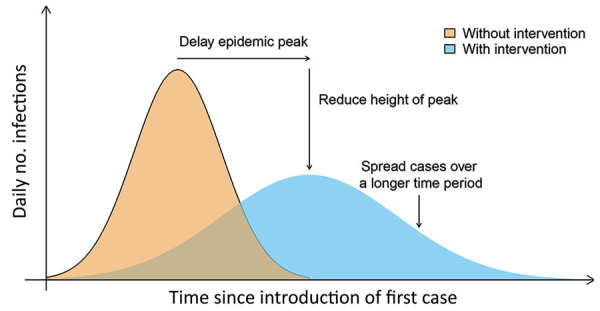
Intended impact of social distancing measures as nonpharmaceutical interventions for an influenza pandemic. Adapted from similar diagrams in the European Centre for Disease Prevention and Control Technical Report (*3*) and the Centers for Disease Control and Prevention Guidance Report (*4*).

Influenza virus infections are believed to spread mainly through close contact in the community (e.g., homes, workplaces, preschool and day care centers, schools, public places), and more frequent and intense contact among children has a particularly major role in transmission (*5*). Social distancing measures aim to reduce the frequency of contact and increase physical distance between persons, thereby reducing the risks of person-to-person transmission. These measures have played a role in mitigating previous pandemics, including the 1918–19 pandemic (*6*,*7*), and are a key part of current pandemic preparedness plans (*8*,*9*). Although a clear biological and epidemiologic rationale supports the potential effectiveness of social distancing measures, there are few opportunities for rigorous controlled trials of community interventions against influenza. Our objective was to review the evidence base for social distancing measures, focusing on the evidence supporting the effectiveness of these measures in reducing influenza transmission in the community.

## Methods and Results

We conducted separate systematic reviews to gather available evidence on the effectiveness of 6 measures in reducing influenza transmission in the community: isolating ill persons; contact tracing; quarantining exposed persons; school dismissals or closures; workplace measures, including workplace closures; and avoiding crowding (Table 1). We retrieved literature from the Cochrane Library, Embase, Medline, and PubMed. Two authors (M.W.F. and H.G.) reviewed the retrieved literature independently for inclusion and synthesis of evidence, and a third author (J.Y.W.) resolved any discrepancies. We were unable to identify randomized controlled trials for the listed social distancing measures. Therefore, we included observational studies (contemporary as well as analysis of archival data from the 1918 pandemic) and simulation studies. We gave greater weight to observational studies than to simulation studies when we inferred the effectiveness of each measure, because assumptions and parameters in simulation studies are more difficult to assess and validate.

**Table 1:** Summary of results for systematic review of literature on nonpharmaceutical interventions for pandemic influenza*

Type of NPI	No. studies identified	Study designs included	Main findings
Isolation	15	Observational, simulation	Isolation has moderate impact in reducing influenza transmission and impact.
Quarantine	16	Intervention study, observational, simulation	Quarantine has general moderate impact in reducing influenza transmission and impact.
Contact tracing	4	Simulation	Combination of contact tracing with other measures (e.g., isolation and quarantine) can reduce influenza transmission and impact; the addition of contact tracing to existing measures might provide only modest benefit but will need substantial resources.
School closure			
Planned holiday	28	Observational	The transmission of influenza decreases during routine school holidays but might increase after schools reopen.
Reactive closures	16	Observational	The effectiveness of reactive school closure varies.
Preemptive closures	13	Observational	Preemptive school closure has moderate impact in reducing influenza transmission.
Workplace measures	18	Intervention study, observational, simulation	Workplace measures are effective; combination with other interventions will further strengthen the effect.
Workplace closures	10	Simulation	Workplace closures might have modest impact in reducing influenza transmission.
Avoiding crowding	3	Observational	Timely and sustained application of measures to avoid crowding might reduce influenza transmission.
*Details of literature review are described in the Appendix.			

### Isolating Ill Persons

We focused on the measure of isolating ill persons at home, but not in medical facilities, because it is unlikely that medical facilities would have the capacity for isolating persons with mild symptoms beyond the early stages of the next pandemic. We reviewed 4 observational studies (*6*,*8**–**10*) and 11 simulation studies (Appendix Tables 3, 4). Outbreaks of influenza A(H1N1)pdm09 during 2009 in various settings, including a navy ship from Peru and a physical training camp in China, have provided evidence that isolating case-patients, together with other personal protective, social distancing, and environmental measures, had substantial effect on reducing attack rates of outbreaks (*8*,*10*). During the 1918–19 pandemic, excess death rates caused by pneumonia and influenza decreased in some cities in the United States after a mixture of interventions were implemented, including isolation or quarantine, school closure, banning of public gatherings, and staggered business hours (*6*).

Although simulation studies were conducted on the basis of a wide range of assumptions, most of these studies suggested that isolation would reduce transmission, including reducing the epidemic size and delaying the epidemic peak. However, Fraser et al. (*11*) discussed the difficulty in controlling influenza transmission, even with high level of isolation combined with contact tracing and quarantine, because of the potentially high proportion of influenza transmission that occurs from mild or asymptomatic infections.

Given that influenza is believed to spread from person to person mostly through close contact, there is a clear rationale for preventing contact between infectious and susceptible persons. However, we found limited scientific evidence to support the effectiveness of this intervention in the community. The observational studies included in this review were conducted in atypical settings, and the effectiveness of isolation in these settings might not be generalizable to the community-at-large. Nonetheless, with the rationale discussed, and assuming that a high level of compliance with home isolation is possible for symptomatic persons, voluntary home isolation could be a preferable strategy to prevent onward transmission compared with other personal protective measures, which have not shown effectiveness in multiple randomized controlled trials.

One area in which there is a lack of evidence is the duration of infectivity, which has implications for the period of voluntary isolation. Current recommendations include voluntary isolation until cessation of fever or until 5–7 days after illness onset (*4*,*12*). The second recommendation would be a better trigger for uncomplicated cases without concurrent conditions, benchmarking the duration of viral shedding (*13*). Another area of uncertainty is the degree to which transmission occurs before illness onset (presymptomatic transmission) and the degree to which mild or asymptomatic cases are infectious. If there is a substantial fraction of asymptomatic transmission (*14*), this fraction would reduce the impact of isolation.

### Contact Tracing

We reviewed 4 simulation studies, all of which found contact tracing to be effective when used in combination with other interventions, including isolation, quarantine, and prophylactic treatment with antiviral drugs (*11*,*15*–*17*). However, Wu et al. (*15*) estimated that the addition of contact tracing to an existing combination of quarantine, isolation, and antiviral prophylaxis measures would only provide modest benefit, while increasing considerably the proportion of population in quarantine and the consequent costs.

Contact tracing requires substantial resources to sustain after the early phases of a pandemic because the number of case-patients and contacts grows exponentially within a short generation time. Therefore, there is no obvious rationale for the routine use of contact tracing in the general population for control of pandemic influenza. However, contact tracing might be implemented for other purposes, such as identification of case-patients in high-risk groups to enable early treatment. There are some specific circumstances in which contact tracing might be more feasible and justified, such as to enable short delay of widespread transmission in small, isolated communities, or within aircraft settings to prevent importation of cases.

### Quarantine of Exposed Persons

We reviewed 1 intervention study (*18*), 5 observational studies (*6*,*19*–*22*), and 10 simulation studies (Appendix Tables 9, 10). Miyaki et al. (*18*) conducted an intervention study in Japan during 2009–2010 involving 2 companies. One company was used as a control; in the other company, a change was introduced in which employees could voluntarily stay at home on receiving full pay when a household member showed development of influenza-like illness (ILI) until days after the symptoms subside. The authors reported a significant reduced rate of infections among members of the intervention cluster (*18*). However, when comparing persons who had an ill household member in the 2 clusters, significantly more infections were reported in the intervention group, suggesting that quarantine might increase risk for infection among quarantined persons (*18*).

Among the observational studies, Li et al. (*20*) estimated that the mandatory quarantine policy in Beijing during the influenza A(H1N1)pdm09 pandemic reduced the number of cases at the peak of the epidemic by a factor of 5 compared with a projected scenario without the intervention, and also delayed the epidemic peak, albeit at high economic and social costs (*20*). Similar to the intervention study in Japan, van Gemert et al. (*21*) reported an increased risk for infection among household contacts who were concurrently quarantined with an isolated person and estimated that the risk for infection increased with a longer duration of quarantine. The evidence base from simulation studies supplemented these findings, and in general, quarantine is suggested to be able to reduce transmission.

In addition, we found some observational evidence for maritime and onboard quarantine. McLeod et al. (*22*) analyzed archival data for the 1918–19 pandemic from the South Pacific jurisdictions and found that strict maritime quarantine delayed or prevented arrival of the pandemic, indirectly reducing the mortality rate compared with that for islands that practiced partial or no maritime quarantine. However, the applicability of these findings is uncertain because maritime travel is uncommon in the 21st century. Conversely, Fujita et al. (*19*) reviewed the onboard quarantine experience at Narita International Airport in Tokyo, Japan, during the influenza A(H1N1)pdm09 pandemic, and reported that the intervention detected few cases and was ineffective in preventing virus entry into the country (*19*).

Overall, we found that the evidence base was weak for home quarantine. In general, the intervention is estimated to be effective. However, being able to identify case-patients and their close contacts in a timely manner can be challenging during the early phase of a pandemic, and impossible for health authorities after the early phase. Quarantine also raises major ethical concerns regarding freedom of movement because the evidence on the effectiveness is limited, providing no solid rationale for the intervention, in addition to restricting movement of some uninfected and noninfectious persons. The increased risks of infection among quarantined persons (*18*,*21*,*23*) further exacerbate the ethical concerns. Therefore, voluntary/self-quarantine is likely to be preferred over mandatory quarantine in most scenarios (*24*). No evidence-based insights or discussions have addressed the optimal duration of quarantine or deactivating trigger. Theoretically, a quarantine duration of 4 days might be sufficient, covering 2 incubation periods of influenza (*25*). If necessary, the duration could be adjusted once the incubation period distribution of the pandemic virus strain is established. Prolonged quarantine can cause substantial burden to social services and working persons (*26*). Some measures can be taken to minimize the possible harms, such as pairing quarantine with antiviral prophylaxis provision for the household (*23*).

### School Dismissals or Closures

School dismissal refers to the situation where a school campus remains open with administrative staff and teachers present but most children stay at home. Schools can then continue to provide meals for children from low-income families or look after children of essential workers. School closure is a stricter intervention in which a school campus is closed to all children and all staff. Although most of the currently available studies on the impact of school dismissals or closures on influenza transmission are presented as studies of school closures, we found that the interventions applied were in some instances school dismissals. Because it was not always possible to identify whether a scenario involved closure or dismissal, and because we expected the effects of closure and dismissal on transmission to be roughly similar, we did not distinguish between the 2 scenarios in our systematic review.

Jackson et al. (*27*) published a systematic review in 2013 that included 79 epidemiologic studies on school closures and found compelling evidence that school closures could reduce influenza transmission, especially among school-age children. However, the duration and the optimal timing of closure were not clear because of the heterogeneity in the available data, and transmission tended to increase when schools reopened (*27*). To update the evidence base presented by Jackson et al., we identified 22 additional studies published since 2013 and included 101 epidemiologic studies in total (Appendix Tables 14–17). Most of these studies were conducted in primary and secondary schools; only a few studies were conducted in universities. Overall, findings from the updated systematic review supported the conclusions by Jackson et al.

Thirteen studies investigated preemptive school closures, in which schools are closed with the aim of slowing transmission in the community (*28*). A correlation analysis between weekly mortality rates and interventions (which included school closure) during the 1918–19 pandemic in cities in the United States estimated that early and sustained interventions reduced mortality rates by <25% (*29*). Two studies conducted in Hong Kong as a public health response to influenza A(H1N1)pdm09 estimated that school closures, followed by planned school holidays, reduced influenza transmission (*30*,*31*).

We found 16 studies reporting the effectiveness of reactive school closures, in which individual schools or groups of schools were closed after substantial ILI outbreaks in those schools (*28*). Two studies conducted in Japan estimated that the peak number of cases and the cumulative number of cases were reduced by ≈24% (*32*) and 20% (*33*). However, some studies estimated that reactive school closures had no effect in reducing the total attack rate and duration of school outbreaks, and the spread of influenza (*34**–**36*).

The effect of routine school holidays in reducing influenza transmission was investigated in 28 studies. Planned school holidays were estimated to reduce influenza transmission and delay the time to epidemic peak occurrence for >1 week (*37*,*38*). In some instances, transmission resurged after schools reopened (*39*).

It is well established that school children play a major role in spreading influenza virus because of higher person-to-person contact rates, higher susceptibility to infection, and greater infectiousness than adults (*40**,**41*). Therefore, school closures or dismissals are a common-sense intervention to suppress transmission in the community, and several observational studies have confirmed that overall transmission of influenza in the community is reduced when schools are closed. However, major caveats are noted in the literature, primarily that transmission will only be reduced when schools are closed. In some past epidemics, closing of schools after the epidemic peak showed little impact on the overall attack rate and none on the timing of the peak or the size of the epidemic peak because it has already passed (*27*). In other past epidemics, transmission resurges after schools reopen, so that the closures delayed the epidemic peak but might not necessarily have reduced the size of the epidemic peak or the overall attack rate (*27*). Although these points seem obvious, the appropriate timing and duration of school closures can be difficult to discern in the heat of an epidemic with delays in information and difficulties in interpreting surveillance data.

School closures can also have adverse impacts on ethical and social equity, particularly among vulnerable groups (e.g., low-income families), which could be ameliorated by dismissing classes, but allowing some children to attend school for free school meals or to enable parents to go to work. Extended school closures might increase domestic travel and contact rates in households and other social gatherings (e.g., malls, theaters), with the potential to increase transmission in the community. The optimum combination of timing, geographic scale, and duration of school closure might differ for the control of different epidemic/pandemic scenarios (*42*). A useful area for further research would be providing validated tools to enable real-time estimation of not only how an epidemic or pandemic is progressing (*43*), but also what the public health impact of an intervention, such as school closure, would be with alternative choices of timing and duration.

### Workplace Measures and Closures

Workplace measures and closures aim to reduce influenza transmission in workplaces or during the commute to and from work. Teleworking at home, staggered shifts, and extended holidays are some common workplace measures considered for mitigating influenza pandemics. A systematic review of workplace measures by Ahmed et al. (*2*) concluded that there was evidence, albeit weak, to indicate that these measures could slow transmission, reduce overall attack rates or peak attack rates, and delay the epidemic peak. We updated the evidence base with 3 additional recently published studies and obtained similar results (Appendix Table 20). Paid sick leave could improve compliance with a recommendation to stay away from work while ill (*44**,**45*).

We conducted a separate search for evidence on the effectiveness of workplace closures in influenza pandemics and identified 10 studies, all of which were simulation studies (Appendix Table 21). In general, the simulation studies predicted that workplace closures would be able to reduce transmission somewhat in the community, but probably would have a smaller effect on transmission than school closures.

We found limited evidence that workplace measures and closures would be effective in reducing influenza transmission. Two recent studies not included in our systematic review have contrasting findings on the effect of having paid sick leave and taking a day off from work because of ILI (*46**,**47*). As with school closures, the timing and duration of workplace interventions would be a critical issue affecting their impact in mitigating a pandemic. This scenario is an area with rich potential for intervention studies to contribute higher quality evidence (e.g., teleworking policies or staggered shifts). However, workplace measures and closures could have considerable economic consequences, and inclusion in pandemic plans would need careful deliberations over which workplaces might be suitable for application of interventions, whether to compensate employees or companies for any loss in income or productivity, and how to avoid social inequities in lower income workers, including persons working on an ad hoc basis.

### Avoiding Crowding

We reviewed 3 observational studies (*6**,**48**,**49*). Timely bans on public gatherings and closure of public places, including theaters and churches, were suggested to have had a positive effect on reducing the excess death rate during the 1918 pandemic in the United States (*6*,*48*). During an influenza outbreak that occurred during World Youth Day 2008, a higher attack rate was reported among a group of pilgrims accommodated in 1 large hall than in pilgrims sleeping in smaller groups (*49*).

The evidence for avoiding crowding is limited. The implementation of measures to avoid crowding might require a large amount of resources (e.g., financial and trained personnel), which might be less feasible in low-income and middle-income countries. Measures to avoid crowding might also be difficult to implement in some settings because of cultural and religious reasons (e.g., Hajj).

## Discussion

Overall, our systematic reviews suggested that social distancing measures could be effective interventions to reduce transmission and mitigate the impact of an influenza pandemic. However, the evidence base for these measures was derived largely from observational studies and simulation studies; thus, the overall quality of evidence is relatively low. Natural experiments or controlled studies of single or combined interventions are needed to clarify the use of social distancing measures; improve knowledge on basic transmission dynamics of influenza, including the role of presymptomatic contagiousness and the fraction of infections that are asymptomatic (*50*); determine the optimal timing and duration for implementation of these measures, and school closures in particular; and provide cost-benefit assessment for implementation of these measures (Table 2).

**Table 2:** Knowledge gaps on social distancing measures as nonpharmaceutical interventions for pandemic influenza and suggested areas for future study

Intervention	Knowledge gaps	Suggested studies
Isolation of sick persons	Few observational studies use laboratory-confirmed influenza as outcome and study isolation as a single intervention; most observational studies were in atypical settings; transmission dynamics of influenza: role of presymptomatic contagiousness, fraction of infections that are asymptomatic, duration of infectivity; optimal strategy for symptomatic persons, trigger to stop isolation	Randomized trials in community settings to evaluate the effectiveness of voluntary isolation against transmission of laboratory-confirmed influenza; epidemiologic studies to understand transmission dynamics of influenza, including symptomatic profiles and duration of infectiousness; compliance of the public with voluntary isolation at home
Contact tracing	Value of adding contact tracing on top of other existing interventions remain unclear; strategy for feasible implementation	Might not be a research priority for pandemic preparedness because of the lack of feasibility of this intervention
Quarantine of exposed persons	Few observational studies use laboratory-confirmed influenza as outcome and provide evidence on the effect of quarantine as a single intervention or the value quarantine adds to existing interventions; transmission dynamics of influenza: fraction of infections that are asymptomatic, possibility of superspreaders; optimal duration of quarantine	Randomized trials in community settings to evaluate the effectiveness of quarantine against transmission of laboratory-confirmed influenza; epidemiologic studies to understand transmission dynamics of influenza including the incubation period and the asymptomatic fraction
School closures	Triggers to close and reopen schools; optimal timing and duration of school closures, taking into account the possible disruptions to the public; compliance of persons of different socioeconomic status; alternative school-based measures, such as staggering lunch breaks and increasing spacing between desks: feasibility and effectiveness	Observational studies on optimal closure triggers and duration, taking into account the possible disruptions brought by school closures; comprehensive review of the acceptance and compliance of the interventions by different subgroups of the population; develop tools to enable real-time estimation of epidemic or pandemic growth, and the effect of implementing closures at different time points of the epidemic/pandemic; while school-based measures were not specifically covered in our systematic review, it would be useful to examine randomized trials of measures to prevent influenza transmission in schools, such as increasing spacing between desks during influenza seasons
Workplace measures and closures	Triggers to close and reopen workplaces; optimal timing and duration of workplace closure, taking into account the possible disruption to the public; alternative workplace measures (e.g., improving teleworking infrastructure, or providing segregated working areas for persons with mild symptoms): feasibility and effectiveness, cost-benefit	Randomized control trials to evaluate the effectiveness of workplace measures (e.g., telework from home, staggered shifts, weekend extension and paid-leave policies) against laboratory-confirmed influenza transmission; studies on optimal triggers, timing and duration for workplace measures and closures, taking into account the possible disruptions caused by workplace measures; cost-benefit analyses of alternative workplace measures
Avoiding crowding	Methods to reduce population density in different settings (e.g., transport hub, mass events, and public places): feasibility and effectiveness	More observational or simulation studies on the alternative methods to avoid crowding in different settings.
Combined interventions	Limited evidence on synergy of alternative interventions or the best combinations of interventions	Policy studies to identify feasible interventions that would complement each other when combined

Although we reviewed the evidence for each NPI individually, it is common for social distancing measures to be implemented in combination. For example, during the 1918 pandemic, multiple NPIs were implemented simultaneously in some cities in the United States, including school closures and public gathering bans (*6*). Although simulation studies have estimated progressively increasing effectiveness as more NPIs are added, we believe that some thought should be given to identifying interventions that would complement each other when combined. Social distancing measures such as school closures and mall closures could be implemented simultaneously to prevent an increase in social contact rates outside schools. School closures could also be paired with teleworking policies to provide opportunities for parents to take care of school-age children at home.

Despite the limitations and uncertainties, social distancing measures will be useful components of the public health response to the next pandemic. Careful consideration of these measures is required when composing pandemic plans, particularly in terms of public compliance and resource planning and distribution. Recommending that ill persons stay at home is probably the most straightforward social distancing measure, and pandemic plans should consider how to enable ill children and employees to stay at home from school or work. For example, health authorities might recommend suspending the usual requirement for doctors’ notes to support absence from school or work. Finally, although our review focused on nonpharmaceutical measures to be taken during influenza pandemics, the findings could also apply to severe seasonal influenza epidemics.

In conclusion, our review found some evidence from observational and simulation studies to support the effectiveness of social distancing measures during influenza pandemics. Timely implementation and high compliance in the community would be useful factors for the success of these interventions. Additional research on transmission dynamics, and research on the optimal timing and duration of school and workplace closures would be useful.

Appendix Additional information on nonpharmaceutical measures for pandemic influenza in nonhealthcare settings—social distancing measures.
