# Supplementary material for: Nonpharmaceutical Measures for Pandemic Influenza in Nonhealthcare Settings—Social Distancing Measures
Source: Emerg Infect Dis. 2020 May;26(5):976–84. doi: 10.3201/eid2605.190995 (PMC7181908; doi:10.3201/eid2605.190995)
Supplement: Appendix — Additional information on nonpharmaceutical measures for pandemic influenza in nonhealthcare settings—social distancing measures. [file 19-0995-Techapp-s1.pdf]

Article DOI: https://doi.org/eid2605.190995

Nonpharmaceutical Measures for Pandemic Influenza in Nonhealthcare Settings—Social Distancing Measures

Appendix

Isolation of Sick Persons

Terminology

Terms relevant to isolation are defined below (Appendix Table 1):

Appendix Table 1. Definition of terms relevant to isolation

Term	Definition
Isolation	“Separation or restriction of movement of ill persons with an infectious disease to prevent transmission to others” (*1*).
Case isolation	“Separation or restriction of movement of ill persons with an infectious disease” at home or in a healthcare facility to prevent transmission to others (*1*,*2*).
Patient isolation	Isolation of ill persons with an infectious disease in a healthcare facility to prevent transmission to others (*2*).
Home isolation	Home confinement of ill persons with an infectious disease (often not needing hospitalization) to prevent transmission to others (*1*,*2*).
Voluntary isolation	Voluntary “separation or restriction of movement of ill persons” in a designated room to prevent transmission to others. This is usually in their own homes, but could be elsewhere (*1*).
Self-isolation	Refer to ‘Voluntary isolation’

Search Strategy

Literature search was conducted using PubMed, MEDLINE, EMBASE, and CENTRAL to identify literature that were available from 1946 through August 4, 2018. No language limit was applied for the literature search, however literatures in languages other than English were excluded during full-text screening. The inclusion criteria is studies reporting the effectiveness of isolation on control of influenza in nonhealthcare settings. No limitation on study design was applied for study inclusion because preliminary works have identified no randomized-controlled trial for this topic. Systematic review and metaanalyses, as well as studies involving clinical settings were excluded. Two reviewers (M.W.F. and H.G.) independently screened the titles, abstracts and full-texts to identify articles for inclusion (Appendix Table 2).

Appendix Table 2. Search strategy for isolation

Search terms	Search date	Reviewers
#1: “patient isolation” OR “case isolation” OR “voluntary isolation” OR “home isolation” OR “social isolation” OR “self-isolation”<br>#2: “influenza” OR “flu”<br>#3: #1 AND #2	5 August 2018	M.W.F., H.G.

Findings

The initial database search yielded 588 articles, of which 70 were selected for full-text screening based on their title and abstract contents. Of these, 56 articles were excluded; main reasons for exclusion of relevant articles include absence of discussion on effectiveness of isolation and focus on healthcare setting. One other study for inclusion was identified through snowball searches. The study selection process is detailed in Appendix Figure 1.

Of the 15 included studies, 4 are epidemiologic studies, comprising of an analysis of historical data from the 1918–1919 pandemic in 43 cities in the United States and 3 outbreak investigations which occurred in an elderly home in France, a training camp in China, and on a Peruvian navy ship respectively (Appendix Table 3) (*3–6*). The remaining 11 are simulation studies (Appendix Table 4 (*7–16*). Isolation was implemented in the outbreaks as a combination with various other interventions such as antiviral prophylaxis and use of a face mask. Isolation was also studied as a single intervention or combined with other interventions in the 11 simulation studies. It is of note that the simulation studies were conducted based on a wide range of assumptions, for example asymptomatic fraction and contact rate reduction brought forth by isolation, hence providing wide-ranging insights on effectiveness of isolation in different scenarios. These included studies focused mostly on reduction of attack rate, epidemic size, transmissibility, and delay in epidemic peak as outcomes-of-interest. All but one study suggested favorable impact of isolation, or combination of isolation with other interventions.

Reduction of Impact

Eight studies suggested decrease in attack rate (AR) brought about by implementation of case isolation (*3,6–8,10–12,14*). An individual-based simulation model for Great Britain and the United States suggested rapid isolation could reduce the cumulative clinical attack rate from 34% to 27% for a pandemic with R_0 2.0, assuming uniform reductions in contact rates in schools, workplaces and households (*7*). Kelso et al. reported similar findings, in which case isolation alone is able to prevent an epidemic (<10% infected) in a 30,000 persons community with R_0 1.5, when 90% of cases are isolated and such measure is implemented within 3 weeks from the introduction of an initial case (*11*). Although isolation alone has been suggested to be more impactful than other interventions, combination with other interventions further improved the effectiveness (*10–12,14*). In addition, increase in isolation rate is quasi-linearly correlated with decrease in attack rate of influenza (*8*).

A reduction in the cumulative incidence of infections due to an isolation policy was also recorded during an influenza A(H1N1)pdm09 outbreak on a navy ship (*6*). A combination of isolating cases of influenza-like illness (ILI), use of masks and hand sanitizers was implemented. The clinical attack rate in the outbreak was 23.9%, a significant reduction from the 97% projected in the absence of any intervention. This also corresponded to a reduction in the effective reproduction number (R) from 1.55 to 0.7 with the intervention. Chu et al. reported similar findings in an outbreak in a physical training camp, in which the final AR recorded was ≈25% of the projected AR of 81% in absence of previous exposure, immunity, and any interventions. In the 1918–19 pandemic, excess death rates due to pneumonia and influenza decreased in New York City and Denver after isolation and quarantine were implemented (*5*).

Conversely, Fraser et al. discussed the difficulty in controlling influenza even with high level of case isolation combined with contact tracing and quarantine, due to the high proportion of asymptomatic transmission of influenza (*9*). The probability of self-isolation without increased public health effort by persons in the community have also been suggested to be high, at 50% and 90% for adult and children respectively (*11*).

Delay of Epidemic Peak

The study of Flauhault et al. suggested that case isolation would have the strongest impact on global spread of a pandemic involving 52 cities compared with air travel restrictions and antiviral treatment, such that isolation of 40% of cases would delay the epidemic by 83 days compared with absence of any intervention (*8*). A combination of isolation of 10% of symptomatic cases with 60% reduction in air traffic on the other hand would delay the start of epidemics in each city by an average of 19 days with considerable case reduction (*8*). The study of Wang et al. study showed similar effect albeit focusing on arrival time of influenza pandemic, in which isolation of a moderate proportion of cases delayed the arrival of the pandemic in a subpopulation for about a month, in the circumstance of high compliance and early implementation (*13*). Delay in response will reduce the effectiveness. Combined intervention with quarantine, school closure, community contact reduction, and personal protective measures further augmented the effect (*12*).

Reduction in Transmissibility

Zhang et al. showed in their simulation studies that isolation of cases can reduce household reproduction number to below one, and compensate delay in antiviral drug distribution by 1 to 2 days. Compliance for isolation has to be much higher to offset longer delays (*15*,*16*). An

outbreak in an elderly home in France reported an abrupt cessation of outbreak after case isolation, antiviral treatment and prophylaxis were implemented (*4*). Reduction in reproduction number was also recorded in the navy ship outbreak previously described, by 54% from 1.55 to 0.7 with a combination of interventions (*6*). The projected reproduction number without isolation of cases was 4.5.

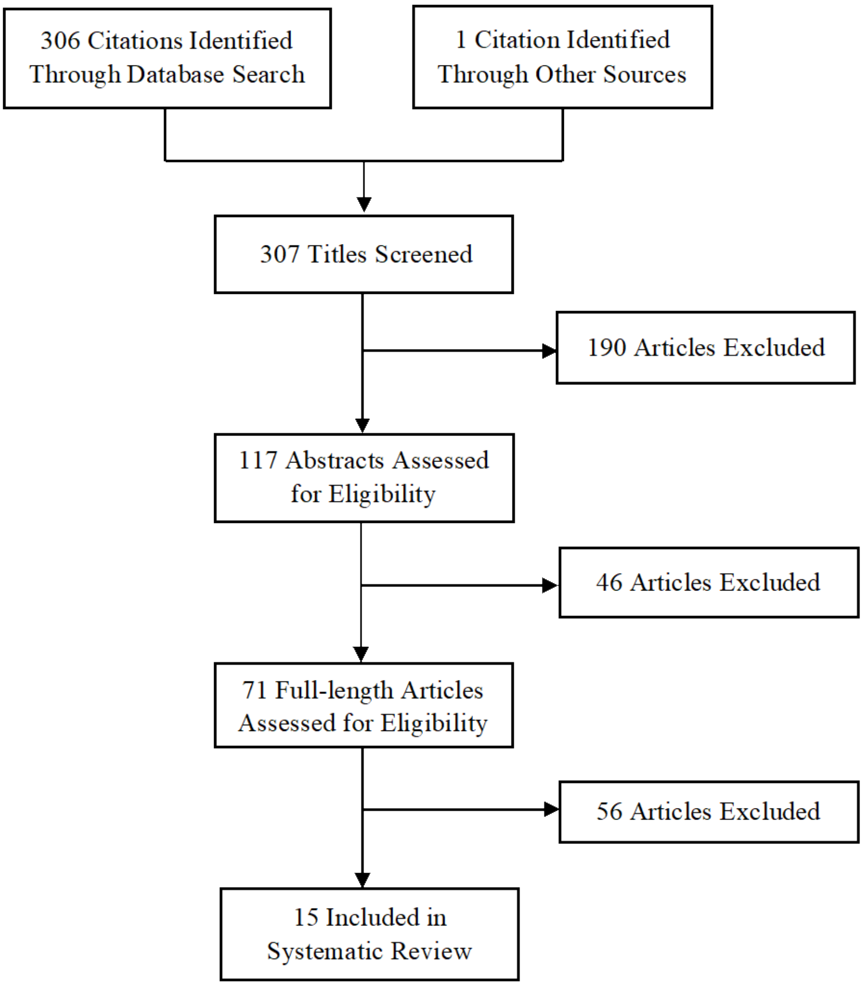

Appendix Figure 1. Flowchart of literature search and study selection for isolation.

Appendix Table 3. Summary of epidemiologic studies included in the review of isolation

Author, year published	Influenza strain or transmissibility (R_0)	Type of study	Study setting and population	Intervention	Comparison	Results and findings
Chu C, 2017 (*3*)	A(H1N1)pdm09	Outbreak investigation	Outbreak in a physical training camp in China with 3256 persons	Combination of isolation with other interventions including oseltamivir treatment and prophylaxis, face-mask usage, cancellation of training and group activities, ventilation and disinfection (implemented within a few days of surge in ILI)	Projected scenario (without previous exposure, immunity and any interventions)	(1) 72.7% clinical cases were reported before intervention, 27.3% after intervention (2) The clinical attack rate recorded for the outbreak was 18.2%, while the projected attack rate in absence of previous exposure, immunity and any interventions was 80.9%
Gaillat J, 2008 (*4*)	Seasonal	Outbreak investigation	Outbreak in elderly home with 81 residents in summer (recorded attack rate of 39.5%)	Sick residents were immediately isolated and used face-masks, oseltamivir treatment and prophylaxis were given to residents and staffs	Not available	No new case was reported among residents and staffs within 2 d of implementation of intervention
Markel H, 2007 (*5*)	1918 pandemic H1N1	Analysis of historical data	43 large cities in the United States; used historical mortality rate data from the US Census Bureau and other historical archival documents	Combination of school closure, public gathering bans, and isolation and quarantine (enforced and mandated respectively)	Cities with different timing, duration and combination of non-pharmaceutical interventions	(1) All 43 cities implemented at least one intervention, 15 cities implemented all three interventions. Cities that started implementation earlier had lower peak mortality and total mortality rates (2). Excess death rate in New York decreased to baseline when isolation and quarantine were implemented, similarly in Denver when school closure, isolation and quarantine were implemented
Vera DM, 2014 (*6*)	A(H1N1)pdm09	Outbreak investigation, stochastic model	Outbreak on a navy ship with 355 crews	Suspected ILI cases were placed in isolation, active case-finding, face mask and hand hygiene, and antiviral provision	Projected scenario (without isolation)	(1) Significant reduction in reproduction number during implementation of interventions (54.4%, from 1.55 to 0.7). The projected reproduction number without isolation was 4.5. (2) Clinical attack rate recorded was 23.9%, while the projected rate was 97%.

Appendix Table 4. Summary of simulation studies included in the review of isolation

Author, year published	Transmissibility of influenza strain (R_0)	Study setting and population	Intervention	Comparison	Results and findings
Flahault A, 2006 (*8*)	3.1 in tropical zone, 0.3–3.4 in other geographic locations due to seasonal variations	(1) Global spread of influenza pandemic from Hong Kong to 52 cities by air trave; (2) Pre-existing immunity in a quarter of the population, 60% of cases are symptomatic	(1) Combination of isolation (10% of symptomatic persons excluded from simulation model) and 60% air traffic reduction (implemented since day 1). (2) Combination of (*1*) with antiviral treatment and vaccination	No intervention	(1) Isolation cause reduction in number of cases by 9%; (2) Cities took on average 19 more days to attain epidemic status when a combination of isolation and air traffic reduction is implemented; (3) Epidemic is delayed by on average 83 d with 40% of case isolation; number of cases ecreased by 65% with a combination of isolation, air traffic reduction, antiviral provision, and vaccination

Author, year published	Transmissibility of influenza strain (R_0)	Study setting and population	Intervention	Comparison	Results and findings
Fraser C, 2004 (*9*)	Upper bound of R_0 was 21	(1) Early stage of disease outbreak in a community with homogenous mixing (2) Proportion of pre-symptomatic transmission is 30%–50%	Isolation of symptomatic persons contact-tracing and quarantine of some persons who were infected before symptomatic persons were isolated; Interventions were implemented without delay. Efficacy of isolation considered were 75%, 90%, and 100%; contact tracing and isolation were assumed to be fully effective.	Not available	Control of influenza is challenging even at high level (90%) of quarantine and contact tracing, due to the considerable proportion of pre-symptomatic transmission.
Halloran ME, 2008 (*10*)	1.9–2.1, 2.4 and 3.0	(1) Model based on population of Chicago (8.6 million persons) with variations in the population structure; (2) 67% infections are symptomatic, case ascertainment levels are 60%–80%	Combination of home isolation (compliance 60/90%; assumed intrahousehold contacts not affected) with quarantine and other social distancing measures, implemented at intervention thresholds of 1, 0.1, and 0.01%	No intervention	At R_0 of 1.9–2.1, 60% ascertainment and 90% compliance, intervention threshold of 0.1%, the attack rate was 0.17%–1.2%, compared with baseline scenario of 42.4%–46.8%
Kelso JK, 2009 (*11*)	1.5, 2.5, and 3.5	(1) Population of 30,000 with contacts in schools, workplaces, other facilities, and between neighboring persons; (2) Asymptomatic fraction mimics that of seasonal influenza	(1) Isolation (assumed no contact outside household, adults and children are 90% and fully compliant respectively; (2) Combination of isolation with school closure, staying away from work and general reduction in community contact	No intervention	(1) An epidemic (≥10% attack rate) at R_0 of 1.5 can only be prevented by case isolation introduced within 3 weeks (as a single intervention), daily attack rate can also decrease from 90/10,000 to <35 if isolation is implemented within a month; (2) Attack rate decreased from 33% to 9% when all 4 measures were implemented together, influenza control is more difficult at higher R_0
Saunders-hastings P, 2017 (*12*)	1.5–2.5	(1) Model based on the population structure of Ottawa–Gatineau census metropolitan area in 2011	Combination of isolation with other interventions including vaccination, antiviral treatment and prophylaxis, school closure, reduction in community contact, personal protective measures, and quarantine; best estimate for compliance for voluntary isolation is 30%	No intervention	(1) Attack rate reduced to 33.9% from the baseline of 53.4% when a combination of isolation and quarantine was implemented, such combination was the most effective among all other interventions studied; (2) Attack rate further reduced to 15.2% and pandemic peak was delayed to more than 100 d when combination of isolation, quarantine, school closure, reduction in community contact and personal protective measures
Zhang Q, 2015 (*16*)	2.5	(1) A community with household distribution based on the Australian census data in 2001; (2) Most infection occur within households and community transmission is negligible	Self-isolation (assumed intra-household contacts remain the same), or combination with antiviral prophylaxis	No intervention	Self-isolation can decrease household reproduction number, compensating the negative impacts of delay in antiviral provision of 1 and 2 d. The compliance for self-isolation have to be considerably higher to compensate for 2 d delay
Zhang Q, 2014 (*15*)	1.5	(1) Stable population with homogenous mixing(2) Asymptomatic fraction is 0.5, and symptomatic cases are 2 times more infectious	Isolation or combination with antiviral prophylaxis	No intervention	(1) Reproduction number decreased to <1 when case isolation is implemented (2) Cumulative number of infections decreased substantially when case isolation is combined with use of antiviral prophylaxis

Author, year published	Transmissibility of influenza strain (R_0)	Study setting and population	Intervention	Comparison	Results and findings
Ferguson NM, 2006 (*7*)	1.4–2.0	(1) Model based on population density and travel behavior data of the U.S. and Great Britain (*2*); 30% of transmission occur in household, the rest in the wider community, workplaces and schools; asymptomatic fraction was 0.5	Rapid case isolation (assumed uniform reduction of contact including household contacts)	No intervention	Cumulative attack rates decreased from the baseline of 34% to 27% for a pandemic with R_0 2.0 if 90% of cases were rapidly isolated
Wu JT, 2006 (*14*)	1.80	(1) Model based on population structure of Hong Kong (i.e. household sizes and average number of children in households); (*2*); 1.5 infected persons introduced each day per 100,000 persons for a year; (*3*); 70% of transmission occur outside household (e.g., in schools and workplaces)	Combination of isolation and voluntary quarantine. Interventions were active before arrival of infected persons in the city.	No intervention	Attack rate decreased from baseline of 74% to 43% when combination of isolation and voluntary quarantine is implemented.
Wang L, 2012 (*13*)	1.75	International spread of influenza to cities during the early phase of a pandemic	Isolation (assumed isolated persons have little chance to cause infection, isolation was implemented by removing some infectious persons from the model)	No intervention	Isolation of a moderate proportion of cases delayed the arrival of the pandemic for about a month, in the circumstance where cases were fully compliant and intervention was started at the first instance of the pandemic
Yasuda H, 2009 (*17*)	A(H1N1)pdm09	Community of 8,800 persons with family structures based on Japanese census data	Home isolation of 1/3 adults and 70%–100% of school-aged children	No intervention	Home isolation of 1/3 adults and all children decreased one-third of the total number of infection

Contact Tracing

Terminology

Contact tracing is the identification and follow-up of persons who may have come into contact with an infected person (*18*). Although contact tracing is often coupled with quarantine or provision of antiviral prophylaxis to exposed contacts, the term contact tracing does not involve these processes.

Search strategy

A literature search was conducted by using PubMed, MEDLINE, EMBASE, and CENTRAL to identify literature available from 1946 to 11 November 2018. No language limit was applied for the literature search; however, literatures in languages other than English were excluded during full-text screening. The inclusion criteria were studies reporting the effectiveness of contact tracing on the control of influenza in nonhealthcare settings. No limitation on study design was applied for study inclusion because preliminary works have identified no RCTs for this topic. Systematic reviews and metaanalyses, as well as studies involving clinical settings were excluded. Two reviewers (M.F. and S.G.) independently screened the titles, abstracts and full texts to identify articles for inclusion (Appendix Table 5).

Appendix Table 5. Search strategy for contact tracing

Search terms	Search date	Reviewers
#1: “contact tracing” OR “trace contact” OR “trace contacts” OR “identify contact” OR “identify contacts” OR “case detection” OR “detect cases” OR “case finding” OR “find cases” OR “early detection”<br>#2: “influenza” OR “flu”<br>#3: #1 AND #2	12 November 2018	M.W.F., H.G.

Findings

The initial database search yielded 1188 articles, of which 75 were selected for full-text screening based on their title and abstract contents. Of these, 71 articles were excluded; the main reasons for exclusion of these articles include absence of discussion on effectiveness of contact tracing and irrelevance. The study selection process is detailed in Appendix Figure 2.

All 4 studies were simulation studies (*9*,*14*,*19*,*20*). None studied contact tracing as a single intervention; instead, this measure was studied in combination with other interventions, such as quarantine, and isolation and provision of antiviral drugs (Appendix Table 6). Such combinations of interventions have been suggested to reduce transmission and delay the epidemic peak (*9*,*14*,*20*).

Reduction of Impact

Wu et al. estimated in their simulation model of an influenza pandemic with a reproductive number (R_0) of 1.8 that the combination of contact tracing, quarantine, isolation and antivirals can reduce the infection attack rate from the baseline of 74% to 34% (*14*). However, the addition of contact tracing on top of quarantine and isolation measures was suggested to provide only modest benefit, while at the same time greatly increasing the proportion of quarantined persons. Conversely, Fraser et al. suggested that it would be difficult to control influenza even with 90% contact tracing and quarantine, due to the high level of presymptomatic or asymptomatic transmission in influenza (*9*).

Delay of Epidemic Peak

In an epidemic of R_0 1.58 in the population structure of Germany, a combination of isolation, treatment of cases, contact tracing, quarantine and postexposure prophylaxis for both community and household contacts, in addition to some household-focused measures, have been estimated to delay the epidemic peak for up to 6 weeks, assuming a case detection rate of 10%–30% (*20*). The authors assumed that the above combination of measures would be 75% effective in reducing secondary cases, and household-focused measures would be 50% effective.

Reduction in Transmissibility

Peak et al. compared the combination of contact tracing with quarantine or symptom monitoring in the early phase of an epidemic with an R_0 of 1.54 (*19*). The study suggested that contact tracing combined with quarantine was more effective than a combination with symptom monitoring in reducing transmission.

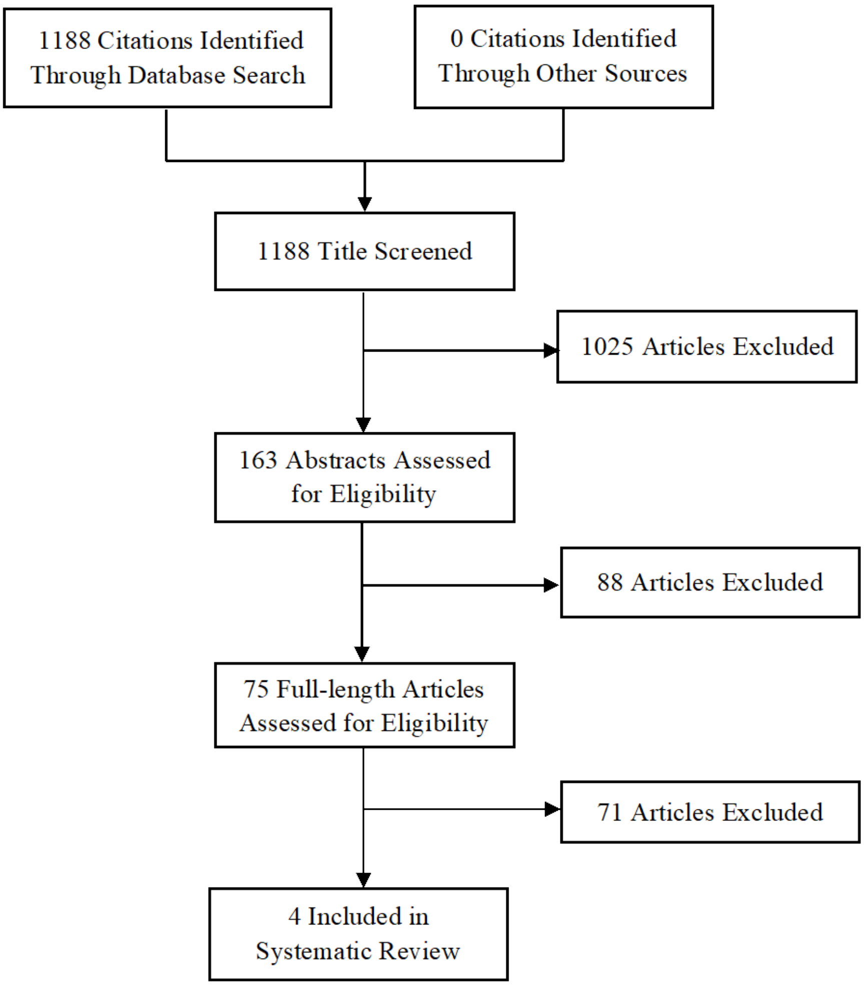

Appendix Figure 2. Flowchart of literature search and study selection for contact tracing

Appendix Table 6. Summary of studies included in the review of contact tracing

Author, year published	Transmissibility of the influenza strain (R_0)	Study setting and population	Intervention	Comparison	Results and findings
Wu JT, 2006 (*14*)	1.80	(1) Model based on population structure of Hong Kong (i.e., household sizes and average number of children in households) (*2*); 1.5 infected persons introduced each day per 100,000 persons for a year (*3*); 70% of transmission occur outside household (e.g., in schools and workplaces)	Combination of contact tracing with other interventions such as quarantine, isolation and antivirals. For contact tracing, persons were asked to name on average five members of their peer group. The contacts of all new symptomatic or hospitalized cases were traced with a mean delay of 1 d. Contacts were asked to take precautionary measures. Interventions were active before arrival of infected persons in the city	No intervention	Attack rate decreased from baseline of 74% to 40% when combination of isolation, quarantine and antivirals is implemented. Addition of contact tracing to the combination of interventions further reduced attack rate to 34%, but increased considerably the proportion of population in quarantine
Peak CM, 2017 (*19*)	1.54	(1) Initial infected population of 1000 persons during the early phase of an epidemic (*2*); no substantial depletion of susceptibles within first few generations of transmission	Symptomatic contacts were isolated immediately, asymptomatic contacts were placed under quarantine (in a high performance scenario, delay in contact tracing was 0.5 ± 0.5 d, 90% of contacts were traced, 50% of traced contacts were infected)	Asymptomatic contacts were placed under symptom monitoring instead of quarantine	Combination of contact tracing with quarantine is more effective in reducing reproduction number compared with combination of contact tracing with symptom monitoring
Fraser C, 2004 (*9*)	Upper bound of R_0 was 21	(1) Early stage of disease outbreak in a community with homogenous mixing (2) Proportion of pre-symptomatic transmission is 30%–50%	Isolation of symptomatic persons, contact-tracing and quarantine of some persons who were infected before symptomatic persons isolated; Interventions were implemented without delay. Efficacy of isolation considered were 75%, 90%, and 100%; contact tracing and isolation were assumed to be fully effective.	Not available	Control of influenza is challenging even at high level (90%) of quarantine and contact tracing, due to the considerable proportion of pre-symptomatic transmission.
an der Heiden M, 2009 (*20*)	1.34, 1.58, 2.04	(1) Model based on the population structure of Germany: 71,000,000 adult and 11,000,000 children (<15 y old), whole population is completely susceptible at the beginning of the epidemic (*2*); Children are 2.06 times more susceptible than adults, 86% of infected persons show development of symptoms	(1) Intensive case-based measures (CCM1; consisting of isolation and treatment of cases, contact tracing, quarantine and post-exposure prophylaxis of some household and community contacts) (*2*); Less-intensive measures (CCM2; isolation and treatment of cases, quarantine and post-exposure prophylaxis of only household contacts); CCM1 and CCM2 were assumed to be 75% and 50% respectively in their effectiveness to reduce secondary cases	No intervention	(1) When the initial 500 cases were subjected to CCM1 and the subsequent 10,000 cases CCM2, the peak of the epidemic is delayed for up to 6 weeks (R_0 1.58, 5 imported cases per day, case detection rate 10%–30%). If only CCM1 was adopted, the delay was estimated to be 6–20 d (case detection rate 10%–30%) (*2*); Effectiveness of these combination of interventions is affected by the R_0 of the influenza strain and case detection rate, i.e., higher R_0 causes interventions to be ineffective at an earlier time point.

Quarantine of Exposed Persons

Terminology

Terms relevant to quarantine are defined below (Appendix Table 7):

Appendix Table 7. Definition of terms relevant to quarantine

Term	Definition
Quarantine	Imposed "separation or restriction of movement" of persons who are "exposed, who may or may not be infected but are not ill," and "may become infectious to others" (*1*).
Household quarantine	Confinement (commonly at home) of non-ill household contacts of a person with proven or suspected influenza (*1*,*2*).
Home quarantine	Home confinement of non-ill contacts of a person with proven or suspected influenza.
Self-quarantine	Voluntary confinement of non-ill contacts of a person with proven or suspected influenza.
Work quarantine	1) Measures taken by workers "who have been exposed and who work in a setting where the disease is especially liable to transmit (or where there are people at higher risk from infection), e.g. people working in elderly homes and nurses in high risk units" (*1*).<br>2) Measures taken by healthcare workers who "chose to stay away from their families when off-duty so as not to carry the infection home" (*1*).
Maritime quarantine	Monitoring of all passengers and crew for a defined period before disembarking from a ship is permitted in a jurisdiction (*21*).
Onboard quarantine	Monitoring of all passengers and crew for a defined period before disembarking from a flight is permitted (*22*). Also known as 'airport quarantine' (*22*).

Search Strategy

A literature search was conducted by using PubMed, MEDLINE, EMBASE, and CENTRAL to identify literature that were available from 1946 through July 23, 2018. Similar to isolation, no limitation on language and study design were applied for the literature search. Literatures in languages other than English were excluded during full-text screening. Studies reporting the effectiveness of quarantine on control of influenza in nonhealthcare settings were included. Systematic reviews and metaanalyses, as well as studies involving clinical settings were excluded. Two reviewers (M.W.F. and H.G.) independently screened the titles, abstracts and full-texts to identify articles for inclusion (Appendix Table 8).

Appendix Table 8. Search strategy for quarantine

Search terms	Search date	Reviewers
#1: "quarantine"<br>#2: "influenza" OR "flu"<br>#3: #1 AND #2	24 July 2018	M.W.F., H.G.

Findings

The initial database search yielded 1873 articles, of which 120 were selected for full-text screening based on their title and abstract contents. Of these, 104 articles were excluded; the main reasons for exclusion of relevant articles include absence of discussion on effectiveness of

quarantine and focus on healthcare setting. The study selection process is detailed in Appendix Figure 3.

The included studies were comprised of 10 simulation studies (Appendix Table 10) (*7,10,12,14,20,23–27*). The epidemiologic studies included 1 modeling study based on pandemic influenza A(H1N1)pdm09 transmission in Beijing (*28*), 2 analyses of historical data (1918–19 influenza pandemic in the United States and South Pacific, respectively) (*5,21*), and 2 observational studies and an intervention study in Japan (Appendix Table 9) (*22,29,30*). Quarantine measures studied include home quarantine, household quarantine, border quarantine as well as maritime quarantine. Quarantine was studied as a single intervention or as a combination with other interventions, commonly with isolation and antiviral prophylaxis. These included studies focused mostly on reduction of attack rate, transmissibility, and delay in epidemic peak as outcomes-of-interest.

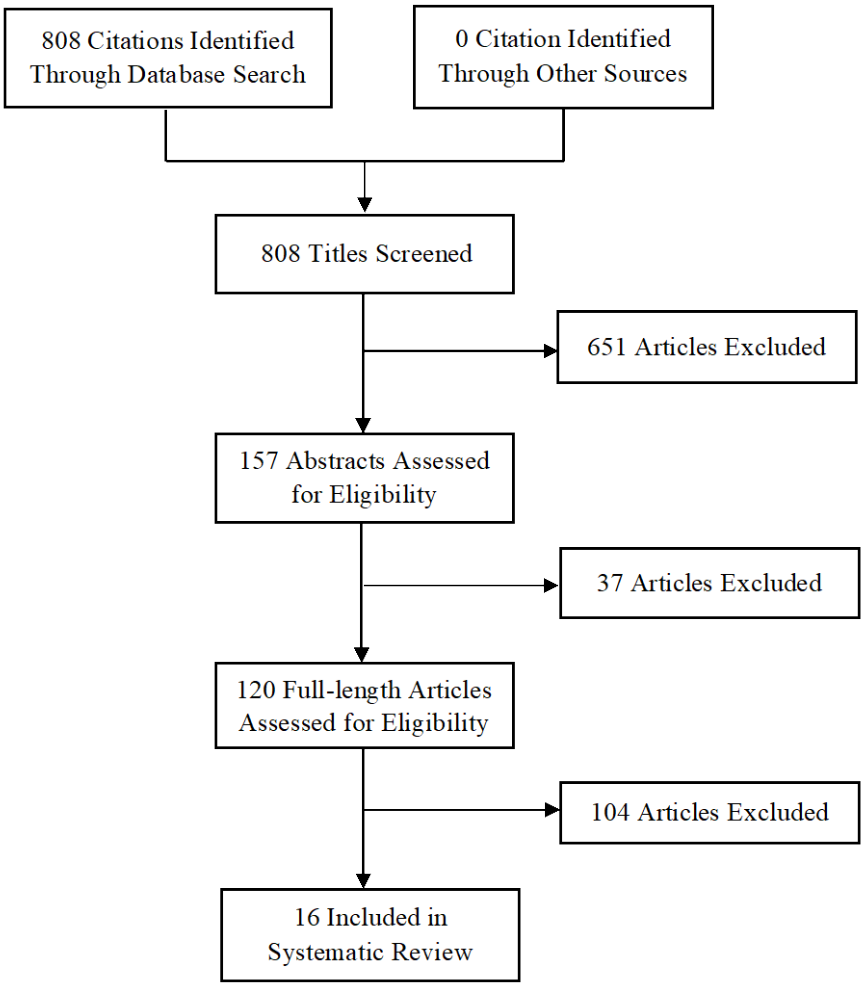

Appendix Figure 3. Flowchart of literature search and study selection for quarantine.

Reduction of Impact

Five studies suggested reduction in attack rate with implementation of household quarantine measures (*7,10,12,14,29*). Miyaki et al. conducted an intervention study in Japan in 2009–2010, which involved 2 companies. Employees of 1 company were used as a control group while in the other company, employees were asked to voluntarily stay at home on full pay if a family member was experiencing ILI. The intervention reduced risk and number of infections for members of the cluster and in the workplace involved (*29*).

Ferguson et al. reported in their simulation study that household quarantine were effective in reducing attack rate at R_0 1–4.2, especially so at low values (*7*). Combination of quarantine with other interventions such as home isolation, provision of antiviral prophylaxis, school closure and workplace distancing were suggested to further reduce the cumulative incidence of infections (*7,10,14*).

Household quarantine has also been suggested to be highly effective in reducing peak and total number of cases in a pandemic, provided that compliance is high (*27*). Longini et al. reported similar findings, that is the effectiveness of household quarantine in reducing number of cases is conditioned by high compliance at 70% and relatively low R_0, in addition to early implementation (*23*). Border quarantine on the other hand has been suggested to cause minimal impact on reduction of number of cases (*26*).

Both analyses of historical data of the 1918–19 pandemic studied the effectiveness of interventions on mortality rates (*5,21*). When a combination of isolation and quarantine was implemented, excess death rates due to pneumonia and influenza decreased in New York City and Denver (*5*). Maritime quarantine in the pacific islands have also delayed or prevented arrival of the epidemic, indirectly reducing mortality rates in the jurisdictions (*21*).

Transmissibility

Both household quarantine and border quarantine have been suggested to reduce transmission, albeit with moderate effectiveness (*22,24,25*). Fujita et al. assessed the onboard quarantine inspection experience in Japan during the 2009 H1N1 pandemic, and reported minimal impact in detecting and preventing entry of cases; however, following-up with passengers thereafter was found to be effective in preventing secondary infection in the community from travelers (*22*). Nishiura et al. also suggested that border quarantine of 9 days would prevent 99% of entry of infectious travelers into small island nations (*24*).

Increased Risk for Household Contacts

Although it showed a reduction of the infection rate in the intervention cluster, the intervention study of Miyaki et al. also reported that more persons became ill in the intervention group when there was an ill family member (*29*). The likelihood of a household contact (concurrently quarantined with an isolated individual) becoming a secondary case has been estimated to increase with each day of quarantine (*30*).

Appendix Table 9. Summary of epidemiologic studies included in the review of quarantine

Author, year published	Influenza strain or transmissibility (R_0)	Type of study	Study setting and population	Intervention	Comparison	Results and findings
Markel H, 2007 (*5*)	1918 pandemic H1N1	Analysis of historical data	43 large U.S. cities; used mortality records from the U.S. Census Bureau and other archival documents	Combination of school closure, public gathering bans, isolation and quarantine (both mandatory)	Cities with different timing, duration and combination of non-pharmaceutical interventions	(1) All 43 cities implemented at least one intervention, 15 cities implemented all 3 together. Cities that started implementation earlier have lower peak and total mortality rates (*2*); Excess death rate in New York decreased to baseline when isolation and quarantine were implemented, similarly in Denver when school closure, isolation and quarantine were implemented
Fujita M, 2011 (*22*)	A(H1N1)pdm09	Observational	Japan (passengers at Narita International Airport for onboard quarantine inspection and Japan at-large for the outbreak)	Onboard quarantine inspection was conducted for over 25 d, on 500 flights carrying 120069 passengers. Cases (identified by thermography screening and positive rapid test) and persons seated around them were isolated. If cases were subsequently confirmed of their infection by PCR, cases were isolated while persons seated around them were quarantined	Not available	Onboard quarantine inspection detected few cases and was ineffective in preventing virus entry into the country. Onboard quarantine however increase the ease to trace and monitor travelers when they are in town, subsequently reduce/ prevent onward transmission in the community.
Li X, 2013 (*28*)	A(H1N1)pdm09	Model based on epidemiologic dynamics of influenza A(H1N1)pdm09	Beijing (N = 20 million); used data of daily confirmed cases reported by Beijing Municipal Bureau of Health (May-July 2009)	Mandatory quarantine for all close contacts	Projected scenario (without mandatory quarantine)	Reduced number of cases at peak of epidemic to 5 times less than the projected scenario in which mandatory quarantine was not conducted, and delayed epidemic peak. Pandemic size remained the same and authors discussed on high economic and social costs of quarantine
McLeod MA, 2008 (*21*)	1918 pandemic H1N1	Analysis of historical data	South Pacific islands (including Australia); used records from national archives of relevant countries, government departments, and international organizations	Maritime quarantine (monitoring all passengers and crew for on average 5–7 d before allowing disembarkation)	Jurisdictions with partial or no maritime quarantine implemented	Strict maritime quarantine have delayed or prevented arrival of the pandemic in said jurisdictions, and associated with reduced mortality rate. Partial quarantine (*i.e.* routine release, no quarantine of asymptomatic passengers) in Fiji and Tahiti was unsuccessful, as in other jurisdictions that did not adopt any border control interventions
Miyaki K, 2011 (*29*)	A(H1N1)pdm09	Intervention study	15,134 general employees (aged 19–72 y) of two sibling companies in japan.	Employees in the intervention cluster were asked to stay home voluntarily on full pay if any household family members showed development of ILI, until 5 d after ILI symptoms	Employees in the control cluster reported to work as usual even when a household member is experiencing ILI	Infection in workplace is significantly reduced among the intervention cluster, however participants in this group are more likely to be infected when there is an infected household member

Author, year published	Influenza strain or transmissibility (R_0)	Type of study	Study setting and population	Intervention	Comparison	Results and findings
				subside or 2 d after cessation of fever.		
van Gemert C, 2011 (*30*)	A(H1N1)pdm09	Retrospective cross-sectional	Confirmed cases reported to the Victorian Department of Health, Australia from May-June 2009 (n = 36 index case-patients, 131 household contacts)	Antiviral drug usage (treatment and prophylaxis) and household quarantine	Not available	The likelihood of a household contact (who was concurrently quarantined with a case) to become infected increase for each additional day of quarantine (adjusted OR 1.25, 95% CI 1.06–1.47)

Appendix Table 10. Summary of simulation studies included in the review of quarantine

Author, year published	Transmissibility of influenza strain (R_0)	Study setting and population	Intervention	Comparison	Results and findings
an der Heiden M, 2009 (*20*)	1.34, 1.58, 2.04	(1) Model based on the population structure of Germany: 71,000,000 adult and 11,000,000 children (<15 y old), whole population is completely susceptible at the beginning of the epidemic (*2*); Children are 2.06 times more susceptible than adults, 86% of infected persons show development of symptoms	(1) Intensive case-based measures (CCM1; consisting of isolation and treatment of cases, contact tracing, quarantine and post-exposure prophylaxis of some household and community contacts) (*2*); Less-intensive measures (CCM2; isolation and treatment of cases, quarantine and post-exposure prophylaxis of only household contacts); CCM1 and CCM2 were assumed to be 75% and 50% respectively in their effectiveness to reduce secondary cases	No intervention	(1) When the initial 500 cases were subjected to CCM1 and the subsequent 10,000 cases CCM2, the peak of the epidemic is delayed for up to 6 weeks (R_0 1.58, 5 imported cases per day, case detection rate 10%–30%). If only CCM1 was adopted, the delay was estimated to be 6–20 d (case detection rate 10%–30%) (*2*); Effectiveness of these combination of interventions is affected by the R_0 of the influenza strain and case detection rate, i.e., higher R_0 causes interventions to be ineffective at an earlier time point.
Saunders-hastings P, 2017 (*12*)	1.5–2.5	(1) Model based on the population structure of Ottawa–Gatineau census metropolitan area in 2011	Combination of quarantine with other interventions including vaccination, antiviral treatment and prophylaxis, school closure, reduction in community contact, personal protective measures, and isolation; best estimate for compliance for quarantine is 15%	No intervention	(1) Combination of quarantine and isolation caused greatest impact in reducing the attack rate among all interventions studied. Attack rate was reduced to 33.9% from the baseline value of 53.4%.<br>(2) Combination of quarantine, isolation, school closure, community-contact reduction and personal protective measures further decreased the attack rate to 15.2% and delayed the epidemic peak to more than hundred days
Ferguson NM, 2006 (*7*)	1.4–2.0	(1) Model based on population density and travel behavior data of the United States and Great Britain (*2*); 30% of transmission occur in household, the rest in the wider community, workplaces and schools; asymptomatic fraction was 0.5	Voluntary household quarantine for 14 d (assumed 50% compliance, contact rates outside household reduced by 75% and intra-household contact rate doubled)	No intervention	Voluntary household quarantine was effective in reducing community attack rate and delaying epidemic peak, in the circumstance of high compliance. A combination of household quarantine and antiviral prophylaxis provision could further strengthen the effect, at the same time alleviate the ethical dilemma due to the increased risk for infection among quarantined persons
Wu JT, 2006 (*14*)	1.80	(1) Model based on population structure of Hong Kong (i.e., household sizes and average	Combination of isolation and voluntary quarantine (household quarantine of on average 7.2–8.2 d). Interventions were	No intervention	Attack rate decreased from baseline of 74% to 43% when combination of isolation and voluntary quarantine is implemented.

Author, year published	Transmissibility of influenza strain (R_0)	Study setting and population	Intervention	Comparison	Results and findings
		number of children in households) (*2*); 1.5 infected persons introduced each day/100,000 persons for a year (*3*); 70% of transmission occur outside household (e.g., in schools and workplaces)	active before arrival of infected persons in the city		
Halloran ME, 2008 (*10*)	1.9–2.1, 2.4 and 3.0	(1) Model based on population of Chicago (8.6 million persons) with variations in the population structure (*2*); 67% infections are symptomatic, case ascertainment levels are 60%–80%	Combination of household quarantine (for 10 d with compliance of 30%, 60% or 90%) with isolation, and other social distancing measures, implemented at intervention thresholds of 1, 0.1, and 0.01%	No intervention	At R_0 1.9–2.1, 60% ascertainment and 90% compliance, intervention threshold of 0.1%, attack rate was 0.17%–1.2%, compared with baseline scenario of 42.4%–46.8%
Sato H, 2010 (*26*)	2.3	(1)Population of 100,000 persons; (2) Cases which was not detected during onboard quarantine inspection caused transmission in the population	Onboard quarantine combined with school closure and home quarantine (with compliance of 10%, 30% and 50%; quarantined persons were assumed to have no contact with infectious persons for 3, 7, or 14 d)	No intervention	The interventions were effective in reducing maximum number of daily symptomatic cases and delaying the epidemic peak. Such effectiveness depend on compliance; low compliance result in low impact. Home quarantine for 14 d starting on day 6, with compliance of 50% was the most effective, which reduced number of cases by 44% and delayed the epidemic peak by 17 d
Longini IM Jr, 2005 (*23*)	1.4	Population of 500,000 persons with population structure based on the 2000 census in Thailand, and social network structure in rural Thailand	Household quarantine; quarantined persons were assumed to have two times more contact with their household and household cluster members	No intervention	Household quarantine alone was effective in reducing number of cases. Early implementation and high compliance is needed for successful intervention
Nishiura H, 2009 (*24*)	1.67	Small island nation with no previous case, 20 aircrafts (with 8000 passengers and crews in total) arrived in the nation before closure of all airports	All incoming passengers and crews were quarantined on arrival and monitored for symptoms. All infected persons who become symptomatic were successfully detected. Isolation and quarantine were completely effective and no secondary transmission within the facilities	No intervention	Quarantine of 9 d can decrease 99% of risks of introducing infectious persons into small island nations. Combination with rapid diagnostic testing can reduce the quarantine period to 6 d
Roberts MG, 2007 (*25*)	2.0	(1) Population of one million persons (2) 67% of infected persons show development of symptoms; asymptomatic persons have 50% infectivity when compared with symptomatic persons	(1) Home quarantine (70% compliance) for 6 d, which prevents 56% of all transmission from those infected within their household. (2) Home quarantine (50% compliance), which prevents 40% of transmission from household contacts (*3*) Combination of home quarantine with school closure, and targeted antiviral prophylaxis	No intervention	Home quarantine alone was effective in reducing the reproduction number, as well as the proportion of population infected. At higher transmissibility, R_0 3.0, only the combination of home quarantine with school closure and targeted antiviral prophylaxis is effective in preventing an epidemic
Yang Y, 2011 (*27*)	1.79	(1) Population of 8382 persons, with population and social structure based on the city of Eemnes	(1) Household quarantine (home confinement at all times with compliance 25%, 50%, 75%, and 100%). (2) Combination of household quarantine with school closure and avoiding social activities; Delay between interventions and outbreak threshold was less than one day	No intervention	At 50% compliance, household quarantine reduced 12.5% and 20.8% of total number of cases and peak cases respectively, as well as delayed epidemic peak. A combination of all 3 interventions did not add much benefit in reducing the total number of cases, however reduced the peak cases by 56%, and delayed the epdemic peak

School Closures

Terminology

Closure of schools include scenarios either when virus transmission is observed in the school, or an early planned closure of schools before influenza transmission initiates. Types of closure are shown in Appendix Table 11 (*31*).

Appendix Table 11. Definition of terms relevant to school closures

Term	Definition
School closure	School is closed to all children and staff.
Class dismissal	School campus remains open with administrative staff and teachers, but most children stay home.
Reactive Closure/ Dismissal	School is closed after a substantial incidence of ILI-related illnesses is reported among children and/or staffs in that school.
Pre-emptive Closure/ Dismissal	School is closed before a substantial transmission among children and staff is reported.

Search Strategy

The latest systematic review to review the effects of school closures on influenza outbreaks was published in 2013 by Jackson et al. (*32*). To update the systematic review, we conducted additional search in PubMed, Medline, EMBASE, and CENTRAL to identify literature available from January 1, 2011 through September 3, 2018. Inclusion criteria included study designs of randomized controlled trials, epidemiologic studies and modeling studies reporting the effectiveness of school closure. Studies that described ≥1 influenza outbreaks, as well as the combination of school closure and other nonpharmaceutical interventions (NPIs) were also included. Modeling studies were included only if they used influenza surveillance data to evaluate the effectiveness of school closure. Modeling studies based on simulated data or on avian influenza virus, studies without school-specific data, and studies published other than full report were excluded. Articles published other than English were also excluded after full-text screening. Two reviewers (H.G. and M.W.F.) independently screened titles, abstracts and full texts to identify the eligible articles (Appendix Table 12).

Appendix Table 12. Search strategy for school closures

Search terms	Search date	Reviewers
#1: “school closure” OR “class dismissal” OR “school holiday” OR “community mitigation” OR “social distancing”<br>#2: “influenza” OR “flu”<br>#3: #1 AND #2	4 September 2018	H.G., M.W.F.

Findings

The most recent systematic review was published in 2013. Jackson et al. identified 79 epidemiologic studies on school closures and summarized the evidence as demonstrating that this

intervention could reduce the transmission of pandemic and seasonal influenza among school-children, but the heterogeneity in the available data illustrated that the optimum strategy (e.g., the length of closure, reactive or pre-emptive closure) remained unclear (*32*). The flowchart of study selection is shown in Appendix Figure 4.

In the additional search to update the systematic review that was published by Jackson et al. in 2013, a total of 287 papers were identified from the 4 databases, and 12 citations were found in other sources, resulting in 299 citations for screening. A total of 101 full-length articles were assessed for eligibility, and 22 additional articles were identified. A total of 101 articles were included in our systematic review. The flowchart of study selection is shown in Appendix Figure 5.

Among the included 101 articles, 16 articles had data on reactive school closures (*33–48*), 13 articles examined preemptive school closures (*5,49–60*), 28 articles examined the impact of regular school holidays on transmission (*45,47,58,61–85*), and 47 articles were related to outbreak reports or teachers' strikes (*86–132*). The basic characteristic of the studies is shown in Appendix Table 13.

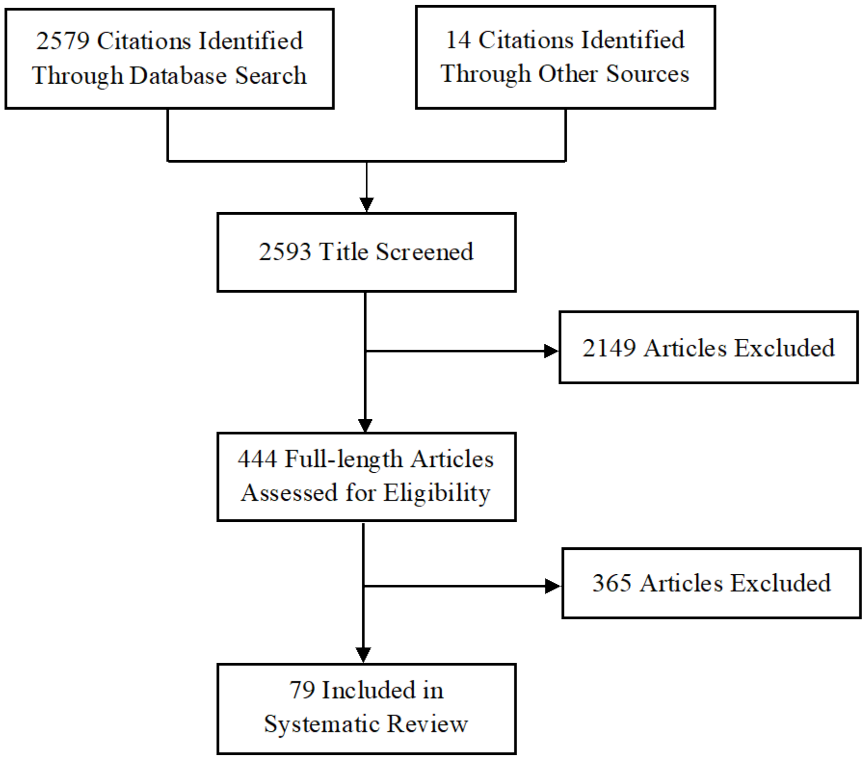

Appendix Figure 4. Flowchart of systematic review by Jackson et al.

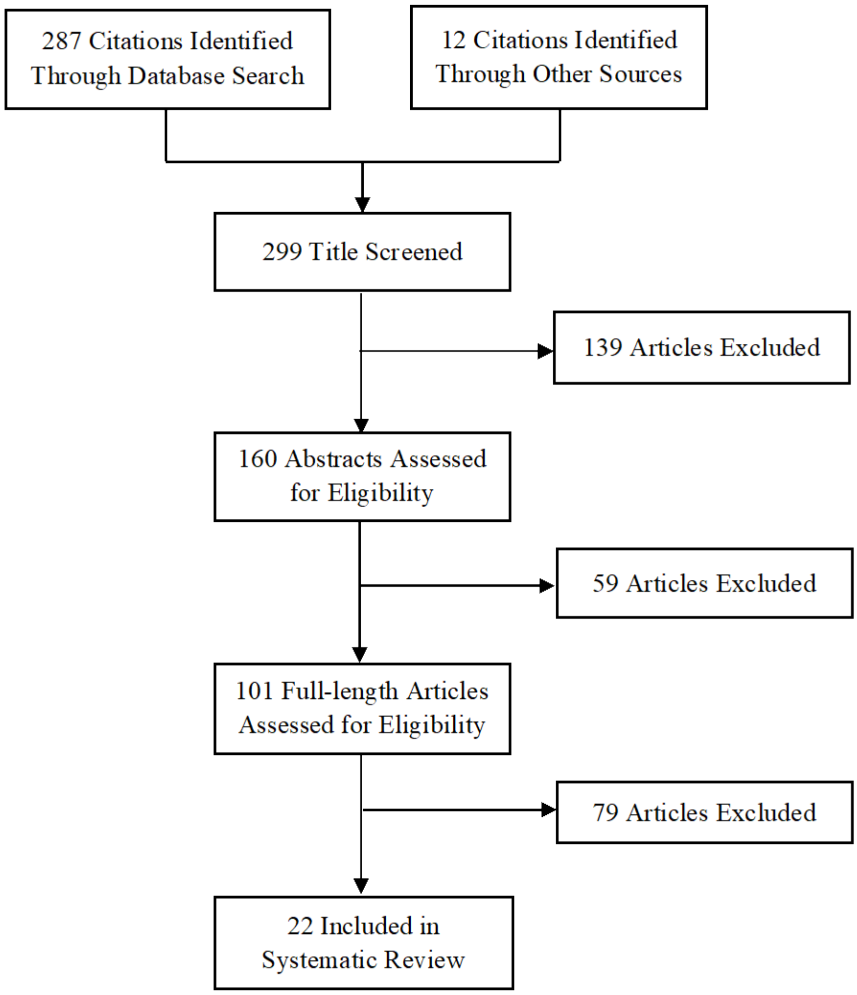

Appendix Figure 5. Flowchart of updated literature search and study selection for school closures.

Appendix Table 13. Basic characteristic of the studies included in school closures

Characteristic	No. studies (n = 101)
Type of influenza strain	
Seasonal	30
1918 pandemic	7
1968 pandemic	1
2009 pandemic	62
Seasonal and 2009 pandemic	1
Study setting	
Asia	30
Europe	26
America	38
Africa	1
Australia	6
Nature of closure*	
Outbreak report or teachers' strike	47
Planned holiday	28
Reactive closure	16
Preemptive closure	13
Duration of closure, d†	
7–13	40
14–20	24
≥21	22
<7	13
Varied	8
Not clear	5

*Articles can contain different nature of closure at the same time
†Each study might have >1 dataset for which the durations of closure differed

Sixteen studies demonstrated that reactive school closure could be a useful control measure during influenza epidemics or pandemics, with impacts that included reducing the incidence and reducing the peak size (Appendix Table 14). Several studies reported a reduction in number of confirmed or ILI cases (*36,37,39,41,45,47,48*). One study also showed a reduction in total infected cases by 32.7% (total reduced number of cases from 127.1 to 85.5) (*44*). Another observational study suggested a reduction in the peak of the epidemic curve by 24% during the 4-day closure and also a reduction of the total number of infected students by 8% (*40*). However, 2 observational studies in China did not identify a significant difference for total attack rate between the control (school closure not implemented) and intervention group (school closed) (*34,35*). Two studies in the United States showed that absenteeism was lower after school reopening compared with before school closure (*42,43*).

The effectiveness of school closures can also be assessed by evaluating the transmission rate (i.e., reproduction number. Hens el al. estimated a reduction of the reproduction number from 1.33 (95% CI 1.11–1.56) to 0.43 (95% CI 0.35–0.52) after school closure (*38*). An observational study from Japan reported that school closure was more effective than class closure (dismissal of that particular class with substantial increase in influenza incidence) (*48*). In another study from Japan, a 2-day school closure in the outbreak situation (after a 10% of absentee occurrence in a school) was associated with the interruption of an outbreak within a week (*46*). One detailed study of transmission in a school in Pennsylvania identified no effect of the reactive closure that was implemented when 27% of students already had symptoms (*33*).

Effectiveness of preemptive school closure was studied in 13 articles (Appendix Table 15). A study showed that preemptive school closure had an advantage to delay the epidemic peak for more than a week, affect the modeled mean peak, and reduce overall attack rate from 9.7% to 8.6% (*49*). Bootsma et al. estimated that early and sustained interventions, including school closures, reduced the overall mortality rate by ≤25% in some cities (*50*). Hatchett et al. (*57*) and Markel et al. (*5*) also examined NPIs during the 1918–19 pandemic and reported that the combined use of NPIs, including school closures, were able to delay the time to peak mortality and to reduce peak and overall mortality rates (*5,57*).

One study estimated a 29%–37% reduction in influenza transmission by the 18-day period of mandatory school closures and other social distancing measures including closure of restaurants and theaters, and cancellation events (*52*). A study in Mexico City estimated that effective reproduction ratio declined from 1.6 before closure to less than 1 during closure (*55*). Wu et al. estimated that the reproduction number was reduced from 1.7 to 1.5 during the pre-

emptive closures and to 1.1 during the rest of the summer holiday (*60*). One study in Mexico showed a 80% reduction of contact rate during closure period and a subsequent planned holiday (*58*). However, closing kindergartens and primary schools for 2 weeks in Hong Kong did not show any significant effect on community transmission, although the incidence remained low after the peak during preemptive closure (*54*).

Twenty-eight studies monitored the change of influenza incidence across planned school holidays, for example the scheduled winter holiday each year, to estimate the impact of school closure on influenza transmission (Appendix Table 16). Of these studies, 8 showed that planned holidays could reduce influenza transmission (*58,61,63,69,70,72,81,85*). One study demonstrated that school holidays reduced the reproductive number R_0 of influenza A(H1N1)pdm09 by 14%–27% in different regions of India compared with a nonholiday period (*61*). One study also reported an association of school holiday with a reduction of 63% to 100% in transmission in Canada (*70*). Another study reported a reduction of R_0 from 1.25 to 0.79 during the 8 days-national holidays in China, but reported that the 8-week summer school holiday had a limited effect on incidence of ILI (*85*). Two studies in the United Kingdom and Mexico showed that school closures could reduce contact rate by around 48%–80% (*58,63*). Two studies in Belgium and the Netherlands suggested that holidays delayed the epidemic peak by >1 week and reduced the peak incidence by 4%–27% (*77,82*). A study from the United States showed that absenteeism in Adrian reduced by ≈6% (*79*), whereas Rodriguez et al. reported no difference between closed schools and those did not close (*80*).

Observational studies also reported a reduction in incidence of influenza associated with planned school holidays (*45,47,62,64–68,71,72,74–76,78,81,83,84*). Studies showed that summer or winter holidays were associated with the reduction of ILI incidences by showing significant changes of ILI incidence rate ratios of school children to adults during the breaks (*65,67,75*). A study based on national surveillance data in France showed that routine school holidays prevented 18% of seasonal influenza cases (18%–21% in children) (*64*). Another study in Japan estimated a 38% reduction in number of medically attended clinical ILI cases (*74*). Wheeler et al. suggested that planned holidays could prevent or delay potential influenza cases among school-age children by ≈42% (*83*). In comparison, a systematic review of simulation studies which review the effects of school closures on influenza outbreaks found that this intervention can be a useful control measure during an influenza pandemic (*133*).

Appendix Table 14. Summary of studies included in the review of reactive school closures

Author, Year	Reduce peak	Reduce overall attack rate	Reduce incidence	Reduce duration	Reduce transmission	Reduce Absenteeism
Cauchemez S, 2011 (*33*)	–	–	–	–	Reproduction number remained unchanged during school closure and after the reopening of school (R = 0.3)	–
Chen T, 2017 (*34*)	–	Total attack rate of 1–3 week of school closure were close to that for no intervention	–	Duration of outbreak was prolonged	–	–
Chen T, 2018 (*35*)	–	Total attack rate of 1–3 week of school closure were close to that for no intervention	–	Duration of outbreak was prolonged	–	–
Davis BM, 2015 (*36*)*	–	–	ILI rate ratio changed from 3.13 (3 weeks before peak), to 2.75 (at peak) and 1.79 (3 weeks after the peak)	–	–	–
Egger JR, 2012 (*37*)	–	–	7.1% reduction in ILI case over the outbreak period	–	–	–
Hens N, 2012 (*38*)	–	–	–	–	Influenza case reproduction number decreased from 1.33 (during outbreak before school closure) to 0.43 (after school closure)	–
Janjua NZ, 2010 (*39*)	–	–	Daily number of ILI cases declined during school closure	–	–	–
Kawano S, 2015 (*40*)^	Number of infected students in a school closure decreased by 24% at its peak	Cumulative number of infected students decreased by 8.0%	–	–	–	–
Loustalot F, 2011 (*41*)	–	–	Incidence remained low during closure	–	–	–
Miller JC, 2010 (*42*)	–	–	–	–	–	Absenteeism was lower after reopening compared with before closure
Russell ES, 2016 (*43*)	–	–	–	–	Closing schools after a widespread ILI activity did not reduce ILI transmission	Absenteeism changed from 1% (baseline), to 3.62% (during school closure), and 0.68% (after school reopening)

Author, Year	Reduce peak	Reduce overall attack rate	Reduce incidence	Reduce duration	Reduce transmission	Reduce Absenteeism
Sato T, 2013 (*44*)	–	Total number of infected persons decreased from 127.1 to 85.5; the maximum number of infected cases decreased from 63.7 to 53.1	–	–	–	–
Sonoguchi T, 1985 (*45*)	–	–	Number of cases declined from 16 on the day before closure to almost 13, 5, and 0 on the three days of closure in high school	–	–	–
Sugisaki K, 2013 (*46*)	–	–	–	Outbreak duration decreased by 4.98 d if the class is closed for 2 d upon the observed 10% ILI-related absentee rate	–	–
Uchida M, 2011 (*47*)			Incidence declined during closure period			
Uchida M, 2012 (*48*)^^	–	–	At elementary school, subsequent peak of H1N1 case showed up despite school or class closure (Figure 1); at junior high school, school closure significantly reduced the number of H1N1 case but not in class closure (Figure 2)	–	–	–

ILI: fever plus cough and/or sore throat
*ILI rate ratio is compared at school district with 51%–100% school being closed vs. district with 1%–50% of school being closed.
^Author mentioned the recommended period of school closure is >4 d
^^Closure duration is significantly related with the number of cases within the 7-d of school opening

Appendix Table 15. Summary of studies included in the review of pre-emptive school closures

Author, Year	Reduce peak	Reduce overall attack rate	Delay time to peak	Reduce incidence	Reduce transmission
Bolton, 2012 (*49*)	–	Overall attack rate decreased from 9.7% to 8.6%*	Epidemic peak would be delayed by over a week	–	–
Bootsma MC, 2007 (*50*)#	Earlier intervention may reduce peak mortality rate	Earlier intervention might reduce total mortality rate	–	–	–
Caley P, 2008 (*51*)#	–	–	–	–	Transmission reduced by 38% during period of social distancing

Author, Year	Reduce peak	Reduce overall attack rate	Delay time to peak	Reduce incidence	Reduce transmission
Chowell G, 2011 (*52*)#	–	–	–	–	Reproduction number decreased from 2.2 (before school closure) to 1.0 (during school closure); transmission rate is estimated to reduce by 29.6% during the intervention period
Copeland DL, 2013 (*53*)	–	–	–	Incidence rate of ARI increased from 0.6% (before closure), to 1.2% (during school closure) and dropped to 0.4% (after school reopening)	–
Cowling BJ, 2008 (*54*)^	–	–	–	–	Not found a substantial effect on community transmission
Cowling BJ, 2010 (*56*)^	–	–	–	–	The estimated reproduction number changed from 1.5 (initial peak) to below 1 (during pre-emptive closure), and fluctuated between 0.8 and 1.3 through the school vacations
Cruz-Pacheco G, 2009 (*55*)#	–	–	–	Incidence increased to peak then decreased gradually during closure period	Effective reproductive ratio R(t) declined from 1.6 before to <1 during closure
Hatchett RJ, 2007 (*57*)#	Earlier intervention reduced peak weekly excess P and death rate	–	–	–	–
Herrera-Valdez MA, 2011 (*58*)#	–	–	–	–	Reduced contact rates by around 80% during closure period
Markel H, 2007 (*5*)#	Earlier intervention reduced peak excess death rate	Earlier and increased duration of intervention reduced total excess death	Earlier interventions increased time to epidemic peak	–	–
Tinoco Y, 2009 (*59*)	–	–	–	Number of ILI cases decreased throughout closure period	–
Wu JT, 2010 (*60*)^					The reproduction number was reduced from 1.7 to 1.5 during the pre-emptive closures and to 1.1 during the rest of the summer holiday

ARI: Presence of at least 2 of the following symptoms: fever, cough, sore throat, or runny nose
ILI: fever plus cough and/or sore throat
#School closure combined with other interventions
^Pre-emptive closure followed by planned holidays
*Assuming schools were closed for 4 weeks and the attack rate in children was 3-fold higher than in adult

Appendix Table 16. Summary of studies included in the review of planned holidays

Author, Year	Reduce peak	Delay peak	Reduce overall attack rate	Reduce incidence	Reduce transmission	Reduce absenteeism
Ali ST, 2013 (*61*)	–	–	–	–	Reproduction number reduced by 14%–27% in different regions of India	–
Baguelin M, 2010 (*62*)	–	–	–	Incidence decreased throughout the closure period	–	–
Birrell PJ, 2011 (*63*)	–	–	–	–	Reduce contact rate among 5–14 y old by 72% (summer holiday) and 48% (half term holiday)	–
Cauchemez S, 2008 (*64*)	–	–	–	Routine school holidays prevented 18% of seasonal influenza cases (18%–21% in children)	–	–
Chowell G, 2011 (*66*)	–	–	–	Number of confirmed cases declined throughout closure period	–	–
Chowell,G, 2014 (*65*)*	–	–	–	Schoolchildren-to-adult ratios decreased by 40%–68% during the 2-week period immediately preceding the winter break	–	–
Chu Y, 2017 (*67*)	–	–	–	ILI incidence rate ratio of children 5–14 years of age (school children) to adult (aged above 60) decreased by 13.3% during summer break	–	–
Davies JR, 1988 (*68*)	–	–	–	Clinical influenza cases increased during closure period	–	–
Eames KT, 2012 (*69*)	–	–	–	–	The initial growth rate of the epidemic during holidays would be 35% lower than during term time (from 1.57 to 1.07)	–
Earn DJ, 2012 (*70*)	–	–	–	–	Reduction in transmission rate in school-age children was 63%, 100% and 86% as a result of schools closing for the summer in Calgary, Edmonton and the Province of Alberta as a whole respectively	–
Evans B, 2011 (*71*)	–	–	–	Estimated number of ILI cases declined during school holiday	–	–
Ewing A, 2017 (*72*)**	–	Figure 5A suggested a peak delay	–	Figure 5B illustrated a reduction of influenza incidence	Influenza transmission decreased by ≈15% (from 1.1 to 0.9) in most seasons	–

Author, Year	Reduce peak	Delay peak	Reduce overall attack rate	Reduce incidence	Reduce transmission	Reduce absenteeism
					and decreased to <1 immediately following Christmas	
Flasche S, 2011 (*73*)	–	–	–	–	No evidence found of a relationship between the effective reproduction number and the start of school holidays	–
Fujii H, 2002 (*74*)	–	–	–	Number of ILI cases decreased by 38% during the first week of closure (from 191 to 118 cases), then increased to 173 cases during the second week of closure	–	–
Garza RC, 2013 (*75*)	–	–	-	ILI incidence rate ratio reduced by 37% among children 5–14 y of age during the week after the winter school break	–	–
Herrera-Valdez MA, 2011 (*58*)#	–	–	–	–	Reduced contact rates by around 80% during closure period	–
Louie JK, 2007 (*76*)	–	–	–	ILI incidence declined throughout closure; laboratory-confirmed declined slightly first, then increased	–	–
Luca G, 2018 (*77*)^	Peak incidence reduced by 4%	All holidays delay the peak time of 1.7 weeks	Epidemic size reduced by ≈2%	–	–	–
Merler S, 2011 (*78*)^^	–	–	–	Incidence decreased during closure	–	–
Monto AS, 1970 (*79*)	–	–	–	–	–	Absenteeism reduced by ≈6% in Adrin
Rodriguez CV, 2009 (*80*)	–	–	–	–	–	No difference in post-break absenteeism in schools on holidays compared with schools that remained open at the same times (relative rate = 1.07, 95% CI = 0.96–1.20)
Smith S, 2011 (*81*)	–	–	–	Consultation rates decreased in school-age children	Transmission of influenza may be interrupted in that school-age group	–
Sonoguchi T, 1985 (*45*)	–	–	–	Case number remained low during closure period in middle school	–	–

Author, Year	Reduce peak	Delay peak	Reduce overall attack rate	Reduce incidence	Reduce transmission	Reduce absenteeism
Te Beest DE, 2015 (*82*)	Epidemic peak is lowered by 27%	Peak is delayed for ≈1 week	–	–	–	–
Uchida M, 2011 (*47*)	–	–	–	Incidence declined during closure period	–	–
Wheeler CC, 2010 (*83*)	–	–	–	Prevent or delay around 42% of potential influenza cases among school age children.	–	–
Wu J, 2010 (*84*)	–	–	–	Cumulative incidence of confirmed cases increased during school closure	–	–
Yu H, 2012 (*85*)	–	–	–	–	Reproduction number changed from 1.25 (before National Day holiday), to <1 (during that holiday), and 1.23 (after that holiday); National day holiday reduced the reproduced number by 37%	–

*Decrease in ratio is caused by a decrease in ILI rates among schoolchildren and the average reduction in ILI incidence among schoolchildren in the 2 weeks during the winter break compared with the 2 weeks before
**The holiday model combined the changes associated with both the school closure and travel models
^All holidays included Fall holiday, Christmas holiday, Winter holiday and Easter holiday
^^Mainly planned holidays, some reactive closures
#School closure combined with other interventions

Appendix Table 17. Summary of outbreak reports and teachers' strike studies included in the review of school closures

Author, Year	Outcome
Armstrong C, 1921 (*86*)	Number of cases peaked on the day following closure and declined thereafter
Baker MG, 2009 (*87*)	Start of the school holidays in New Zealand reduced influenza transmission and that the return to school slightly accelerated the epidemic.
Briscoe JH, 1977 (*88*)	Number of clinical cases declined during closure
Calatayud L, 2010 (*89*)	Cases decline after the half way of school closure
Carrillo-Santisteve P, 2010 (*90*)	Number of confirmed and probable cases declined during closure
Cashman P, 2007 (*91*)	A planned school closure may have contributed to controlling the outbreak without quantitative information
Chieochansin T, 2009 (*92*)	Laboratory confirmed cases declined throughout period of closure
Cohen NJ, 2011 (*93*)	Number of respiratory illness cases were lower on the first day of closure compared with previous days, increased during closure and then declined.
Danis K, 2004 (*94*)	Number of ILI cases declined during closure period
Echevarria-Zuno S, 2009 (*95*)	Epidemic continued while schools were closed and peaked around 1 week after closure
Effler PV, 2010 (*96*)	Number of confirmed cases declined during closure period
Engelhard D, 2011 (*97*)	ILI rate peaked and declined during closure
Farley TA, 1992 (*98*)	Absenteeism remained low after school reopening
Glass RI, 1978 (*99*)	School absenteeism was lower after the holiday than before
Gomez J, 2009 (*100*)	Number of pneumonia cases decreased from 130 cases at peak to around 40 during closure
Grilli EA, 1989 (*101*)	During the mid-term break there were a further 15 ILI cases (daily cases not provided)
Guinard A, 2009 (*102*)	No further cases during school closure period, but epidemic appear to be over before the school was closed
Health Protection Agency West Midlands H1N1v Investigation Team, 2009 (*103*)	Confirmed number of cases declined during closure period
Heymann A, 2004 (*104*)*	Significant decreases in the rate of diagnoses of respiratory infections (42%), visits to physician (28%) and emergency departments (28%) and medication purchases (35%)
Heymann AD, 2009 (*105*)*	Decease in ratio of 14.7% for 6–12 y old associated with teachers' strike
Hsueh PR, 2010 (*106*)	Number of class suspensions or school closure generally associated with the number of hospitalizations
Huai Y, 2010 (*107*)	Number of confirmed cases peak at 30 cases on the first day of closure, then declined during closure period
Janusz KB, 2011 (*108*)	Absenteeism changed from 8% (baseline), to 15% (2 d before school outbreak), and 13% (post-school outbreak)
Johnson AJ, 2008 (*109*)	Number of parentally-reported ILI cases decline because of school closure
Jordan EO, 1919 (*110*)	Incidence declined from 19 cases to 15 cases the following week in elementary school, and declined from 16 to 5 cases in high school
Kawaguchi R, 2009 (*111*)	Number of confirmed cases declined throughout closure period
Lajous M, 2010 (*112*)	Planned holiday was followed by a slight decrease in ILI case numbers
Leonida DDJ, 1970 (*113*)	Absenteeism continued decline during second school closure
Lessler J, 2009 (*114*)	Both confirmed H1N1 influenza and self-reported ILI declined through closure period
Leung YH, 2011 (*115*)	Number of laboratory-confirmed cases increased during first two days of closure and then declined
Lo JY, 2005 (*126*)	Change in proportion of positive specimens were 50%–100% lower in April-June than the average because of community control measures
Marchbanks TL, 2011 (*116*)	Number of ILI cases increased during first two days of closure and then declined
Miller DL, 1969 (*117*)	In children aged 5–14 y, rates of influenza declined during the Christmas holidays
Nishiura H, 2009 (*118*)	Number of laboratory confirmed cases declined throughout the closure
Olson JG, 1980 (*119*)	School absenteeism (all-cause) declined in Girls Teachers' Colleges Primary School; absenteeism very similar before and after closure in Taipei American School
Paine S, 2010 (*120*)	Case numbers peaked and declined during holiday, effective reproduction number declined before holiday and continued to decrease during the holiday
Petrovic V, 2011 (*121*)	Weekly incidence rate of ILI and the number of hospitalized cases decreased after the school closure
Poggensee G, 2010 (*122*)	Practice index was associated with vacation density
Rajatonirina S, 2011 (*123*)	Only few cases continued to occur during closure period
Shaw C, 2006 (*124*)	Absenteeism was lower after closure than before closure in both reactive closure and planned holiday
Shimada T, 2009 (*125*)	Number of new confirmed cases decreased after school closures
Smith A, 2009 (*128*)	Number of ILI cases decreased during closure period
Strong M, 2010 (*129*)	Number of self-reported ILI cases decreased during closure period
van Gageldonk-Lafeber AB, 2011 (*130*)	Possible reduced incidence, or slowed epidemic growth
Wallensten A, 2009 (*131*)	Absenteeism almost not changed before and after closure
World Health Organization, 2009 (*127*)	School absenteeism in the following weeks did not increase after school reopening
Winslow CEA, 1920 (*132*)	Cities with school closures had higher deaths rates; timing and duration of closure were not stated

*Articles related to teachers' strike

Workplace Measures and Closures

Terminology

Workplace measures refers to the methods which can reduce influenza transmission in the workplace, or on the way to and from work, by decreasing frequency and length of social interactions. Workplace closure is the closure of workplaces when virus transmission is observed in the workplace, or an early planned closure of workplaces before influenza transmission initiates.

Search Strategy

The latest systematic review to review the effects of workplace measures in reducing influenza virus transmission was published by Ahmed et al. in 2018 (*134*). To update the systematic review, we conducted additional search in PubMed, Medline, EMBASE, and CENTRAL to identify literature available from January 1, 2017 through September 27, 2018. Workplace measures include teleworking, flexible leave policies, working from home, weekend extension, staggered work shifts, and social distancing at workplaces. All randomized controlled trial, epidemiologic study or simulation study in nonhealthcare workplaces were included in this review. Reviews, commentaries, editorial articles, studies on workplace closure, and studies on generic social distancing irrelevant to workplace were excluded from our review. The following outcomes were extracted from the studies: cumulative attack rate, peak attract rate, occurrence of peak, and others. Two reviewers (H.G. and J.X.) worked independently (Appendix Table 18).

For workplace closure, PubMed, Medline, EMBASE, and CENTRAL were searched to identify literature available from 1946 through September 17, 2018. No language limits were applied to the literature search but papers in languages other than English were excluded in screening. The inclusion criteria included randomized controlled trials, epidemiologic studies and simulation studies reporting the effectiveness of workplace closure in nonhealthcare settings, as well as the combination of workplace closure and other NPIs. The exclusion criteria included the following: studies in healthcare settings; studies that do not have specific data related to workplace closure; reviews, letters, news or summary articles; studies related to avian influenza. Two reviewers (H.G. and E.S.) independently screened titles, abstracts and full texts to identify eligible articles (Appendix Table 19).

Appendix Table 18. Search strategy for workplace measures

Search terms	Search date	Reviewers
#1: “telework” OR “leave” OR “social mixing” OR “social distancing” OR “community mitigation” OR “non-pharmaceutical” OR “nonpharmaceutical”<br>#2: “influenza” OR “flu”<br>#3: #1 AND #2	28 September 2018	H.G., J.X.

Appendix Table 19. Search strategy for workplace closures

Search terms	Search date	Reviewers
#1: "workplace" OR "work site" OR "business" OR "organization" OR "office"<br>#2: "closure" OR "close"<br>#3: "influenza" OR "flu"<br>#4: #1 AND #2 AND #3	18 September 2018	H.G., E.S.

Findings

The most recent systematic review was published in 2018, in which Ahmed et al. (*134*) identified 15 epidemiologic or simulation studies (from 14 articles) on workplace measures. In the additional search, 81 articles were identified from the databases and 1 article from other sources, resulting in 82 articles for title screening. Ten full-length articles were assessed for eligibility, and 3 additional articles were identified (Appendix Table 20). A total of 18 studies (17 articles) were included in our systematic review. The flowcharts of study selection are shown in Appendix Figures 6, 7.

There were 6 epidemiologic studies among the 18 included studies (*29*,*135–139*). A cross-sectional study interviewed randomly selected US adults from the Knowledge Networks online research panel, and showed that persons who cannot work from home (for 7–10 days) were more likely to have ILI symptoms compared with those who could (*135*). Another cohort study suggested that respondents who could work from home had a 30% lower rate of attending work with severe ILI symptoms compared with employees who cannot, suggesting work from home may be able to reduce employee-to-employee transmission (*137*). A cohort study in Singapore estimated that enhanced surveillance and segregation of work units into smaller working subgroups had significantly lower serologically confirmed infections compared with subgroups using the standard pandemic plan (17% vs 44%) (*136*). An intervention study evaluated the effectiveness of voluntary waiting at home on full pay against influenza A(H1N1)pdm09 transmission in workplaces showed an overall risk reduction by 20% (*29*). Piper et al. (*139*) and Asfaw et al. (*138*) used the data from nationally representative survey in the United States and showed that adults with paid sick days had higher probability of staying at home and thus reduced face-to-face transmission in the workplace. The remaining 12 studies were simulation studies reviewed by Ahmed et al. (*134*),and suggested that workplace measure alone reduced the cumulative attack rate by 23%, as well as delaying and reducing the peak influenza attack rate (*10*,*11*,*140–148*).

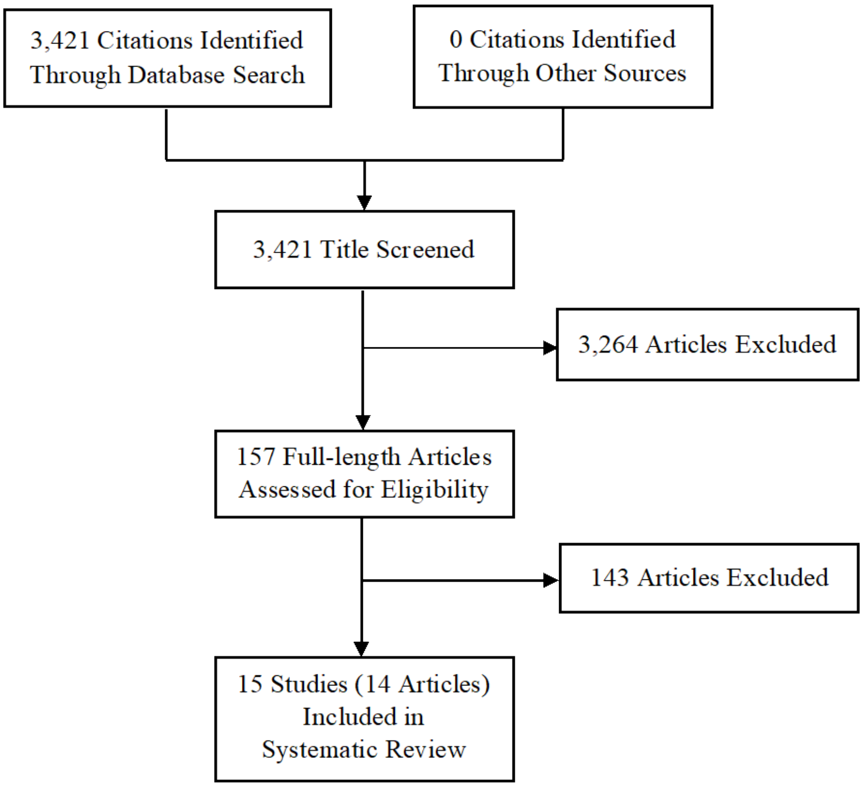

Appendix Figure 6. Flowchart of systematic review by Ahmed et al. (*134*).

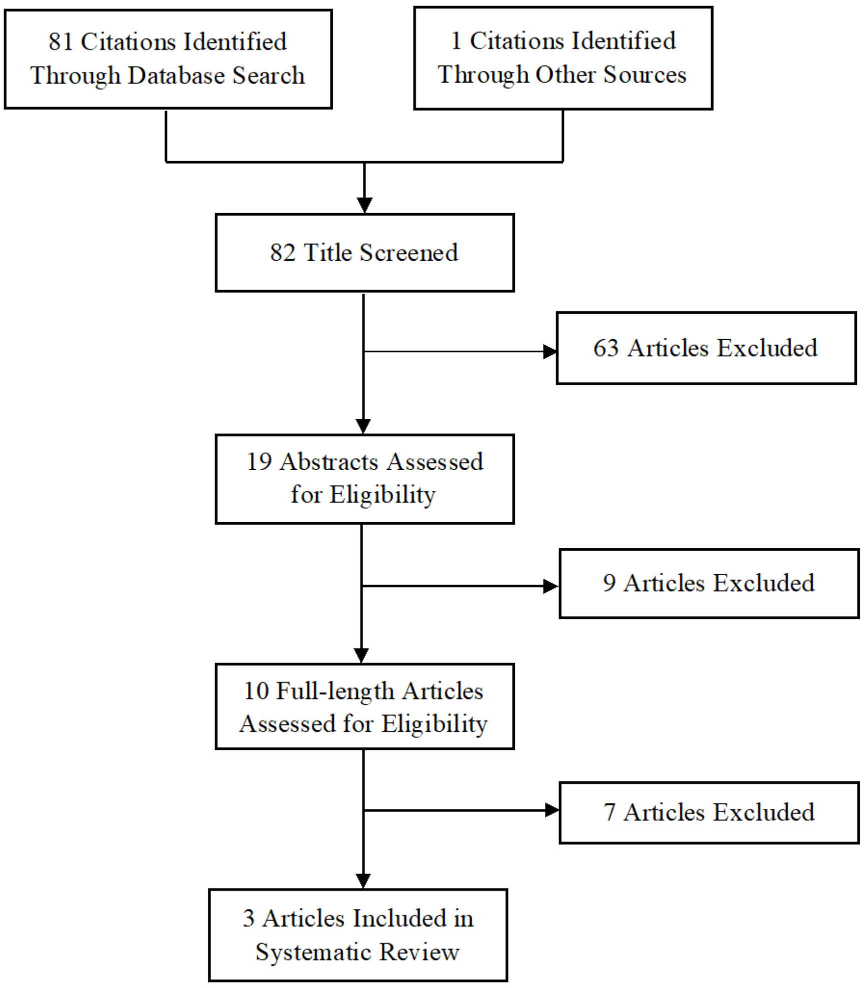

Appendix Figure 7. Flow chart of updated literature search and study selection for workplace measures.

Appendix Table 20. Summary of updated studies included in the review of workplace measures*

Study	Study design	Population and setting	Intervention	Comparison	Outcome
Asfaw A, 2017 (*138*)	National representative survey	Approximately 71,200 persons in the United States	Single: PSL	Without PSL	Employees with PSL had a 32% higher probability to stay at home than workers without PSL, which might benefit the reduction of transmission of influenza
Miyaki K, 2011 (*29*)	Intervention study	Two sibling companies (Cohort 1 n = 6,634, Cohort 2 n = 8,500) in Kanagawa Prefecture, Japan	Single: Voluntary waiting at home on full pay if a household member showed development of ILI	Continue to work in office even when a family member showed development of ILI	Intervention could reduce around 20% overall infection risk in the workplace
Piper K, 2017 (*139*)	National representative survey (3 rounds of interviews in 2009)	12,044 employees over 16 y old in the United States	Single: PSL	Without PSL	Persons with PSL were more likely to stay at home

*ILI, influenza-likw illness; PSL, paid sick leave

For workplace closure, 478 citations were identified through database search and other sources, of which 21 full-length articles were assessed for eligibility and 10 articles were selected for this systematic review. The flowchart of study selection is shown in Appendix Figure 8.

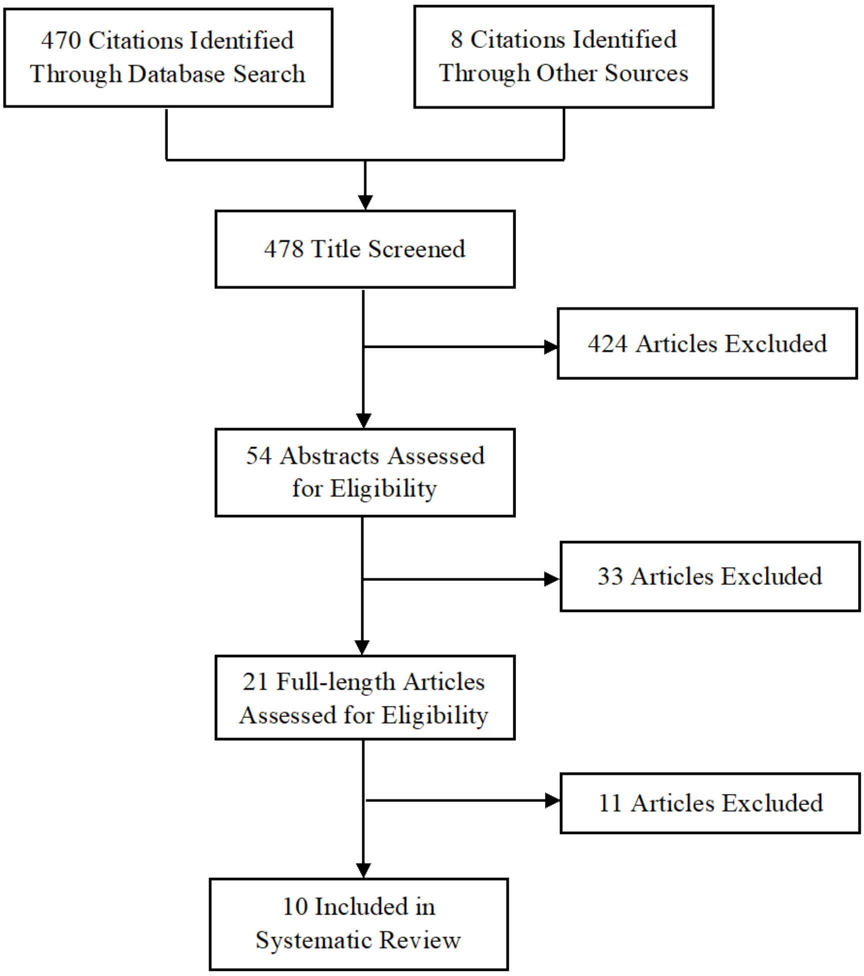

Appendix Figure 8. Flowchart of literature search and study selection for workplace closures.

Among these 10 studies, 8 of them studied combination of workplace closure with school closure, 1 targeted different single and multiple intervention strategies, and 1 evaluated the effectiveness of workplace closure alone (Appendix Table 21). All 10 studies were simulation studies and the main outcomes include the reduction of attack rate, peak number, and delay of epidemic peak.

Predicted Effects reduction

Most included studies suggested the reduction in attack rate, duration of infection or maximum case number. In the studies by Ferguson et al. (*7*) and Xia et al. (*149*), workplace closure resulted in a small reduction in cumulative attack rate, and Carrat et al. (*150*), Mao et al. (*151*), and Halder et al. (*152*) suggested an obvious decrease when assessing the effect of

combined interventions. A study by Carrat et al. simulated individual and community level model in France suggested a decrease of cumulative attack rate from 46.8% to 1.1%, assuming the basic reproduction number (R_0) of 2.07 (*150*). Mao et al. used an agent-based stochastic simulation model with R_0 1.3–1.4 in the United States and predicted a decrease of overall attack rates from 18.6% to 11.9% with 100% school closure (SC) and 10% workplace closure (WC), and from 18.6% to 4.9% with 100% SC and 33% WC (*151*). In addition, a study in Italy suggested that combining strategies including vaccination, prophylaxis and closure of schools, workplaces and public places could reduce the incidence from 50% to ≈15% (*153*).

However, a heuristic model using R_0 of 1.7 and 2.0 suggested a small reduction in cumulative attack rate but a more substantial reduction in peak attack rates (≤40%) when 100% SC and 10% WC was implemented. It also suggested that the effectiveness could increase if 50% of workplaces were closed, at the same time resulting in a higher economic cost (*7*). A simulation model for the control of influenza in an isolated geographic region by Roberts et al. suggested that workplace closure as a single intervention could not prevent the epidemic (R_0 = 2.0) (*25*).

Delay the Time of Peak Occurrence

A simulation study using individual-based model suggested that nationwide closure of schools and workplaces for weeks would delay the time of peak occurrence by 5–8 days, and the effectiveness varied with the R_0 used (1.4, 1.7, and 2.0) (*154*). Rizzo et al. suggested implementing a combination of social distancing measures starting at 4 or 8 weeks of the beginning of a pandemic could delay the peak occurrence by 1 or 3 weeks (*155*). However, a study by Mao et al. estimated that 100% SC and 33% WC could speed up the peak by ≈1 week (*151*).

Appendix Table 21. Summary of studies included in the review of workplace closures*

Study	Influenza strain and transmissibility (R_0)	Study setting and population	Study design	Closure duration	Closure proportion	Closure threshold	Intervention	Comparison	Outcome
Carrat F, 2006 (*150*)	Future pandemic strain; R_0 = 2.07	General population in France (n = 10,000)	Simulation both individual and community level	NA	NA	5 infections/1,000 persons	SC + WC	No intervention	Mean accumulation infection rate reduced from 46.8% (42.3%–50.5%) to 1.1% (0.6%–2.1%)
Ciofi degli Atti ML, 2008 (*154*)	Future pandemic strain; R_0 = 1.4, 1.7, 2	General population in Italy (around 57 million)	Global SEIR model for importation of cases with an individual based model	4 weeks	NA	NA	SC + WC	No intervention	Nationwide closure could delay the peak occurrence by 5–8 d based on various scenarios
Ferguson NM, 2005 (*156*)	Future pandemic strain	Simulated population in Thailand	Stochastic, spatially structured, individual-based discrete time simulation model	NA	NA	NA	SC + WC + antiviral prophylaxis	NA	Interventions could eliminate the pandemic if R_0 is below 1.8
Ferguson NM, 2006 (*7*)	Future pandemic strain; R_0 = 1.7, 2.0	300 million in USA, 58.1 million in UK	Heuristic model	NA	Varied: 10%, 50%	NA	100% SC + varied WC (10%, 50%)	No intervention	100% SC + 10% WC could slightly reduce the cumulative attack rate, and might reduce the peak attack rate up to 40%. 50% of WC could further improve the effectiveness, albeit with a higher economic cost
Halder N, 2011 (*152*)	Future pandemic strain with H1N1 2009 characteristics; R_0~1.3	Albany, Western Australia (n = 30,000)	Individual-based simulation model	Varied: 2 weeks or 4 weeks or continuous	50%	NA	1) SC 2 weeks + 50% WC 2 weeks<br>2) SC 2 weeks + 50% WC 4 weeks<br>3) Continuous SC + 50% WC	No intervention	The three interventions reduced the attack rate by 34.5%, 37.4% and 79.7% respectively
Mao L, 2011 (*151*)	Future pandemic strain; R_0 = 1.3–1.4	Urbanized area of Buffalo, NY, USA (n = 985,001)	Agent-based stochastic simulations	NA	Varied: 10%, 33%	NA	1) 100% SC + varied (10%, 33%) WC;<br>2)100% SC + varied (10%, 33%) WC + preventive behavior	No intervention	1) Overall attack rates declined from 18.6% to 11.9% (10%WC) and 4.9% (33% WC) respectively<br>2) Overall attack rates reduced to 3.99% (10%WC) and 1.83% (10%WC) respectively
Merler S, 2006 (*153*)	Future pandemic strain; R_0 = 1.7	Central Italy (n = 12,489,619)	Individual-based simulation model	4 weeks	NA	20 symptomatic cases were detected	Vaccination + Prophylactic antiviral	No intervention	The incidence dropped from 50% to ≈15%

Study	Influenza strain and transmissibility (R_0)	Study setting and population	Study design	Closure duration	Closure proportion	Closure threshold	Intervention	Comparison	Outcome
							treatment + Quarantine measures (SC + WC + public places)		
Rizzo C, 2008 (*155*)	Future pandemic strain; R_0 = 1.8	National population in Italy (n = 56,995,744)	SEIR deterministic model with a stochastic simulation component	4 weeks	NA	2, 4, or 8 weeks after the start of the pandemic	Nationwide closure of all schools, public offices, and public meeting places	No intervention	Social distancing measures were not effective in reducing attack rate, but could delay the peak occurrence by 1–3 weeks
Roberts MG, 2007 (*25*)	Future pandemic strain; R_0 = 1.1, 2.0 and 3.0	Isolated geographic region (n = 1,000,000)	A model based on published parameters	N/	70%	NA	1) WC; 2) WC + SC; 3) WC + SC + antiviral treat + 70% home quarantine	No intervention	The single strategy of WC is not successful, the combination of all four strategies might prevent the epidemic
Xia H, 2015 (*149*)	Simulate H1N1; R_0 = 1.35, 1.40, 1.45, 1.60	Delhi, India (over 13 million)	Realistic individual-based social contact network and agent-based modeling	3 weeks	60%	Over 0.1% population are infected	Single WC	No intervention	Intervention could reduce the attack rate, peak number, and delay the time of peak occurrence. WC as a single intervention is the most ineffective method among vaccination, antiviral usage, SC, and WC

*NA, not available; SC, school closures; WC, workplace closures.

Avoiding Crowding

Terminology

Avoiding crowding refers to the measures to reduce influenza transmission in crowded areas (e.g., large meetings, conferences, and religious pilgrimages, national and international events).

Search Strategy

Literature available from 1946 through October 17, 2018 were identified from PubMed, Medline, EMBASE, and CENTRAL. Two reviewers (S.G. and E.S.) screened each title, abstract and article that fully met the criteria (Appendix Table 22). Both epidemiologic and simulation studies relevant to the effectiveness of avoiding crowding (e.g., cancellation or postponement of events and limitation of attendance) in public area are included. Studies that only reported outbreak events in a crowded area or perceptions on mass gathering without specific data related to the effectiveness of avoiding crowding; and reviews, letters, news, or summary articles were excluded.

Appendix Table 22. Search strategy for avoiding crowding

Search terms	Search date	Reviewers
#1: “event” OR “meeting” OR “sport” OR “concert” OR “pilgrimage” OR “park” OR “conference” OR “mass” OR “public” OR “community” OR “large” OR “general” OR “church”<br>#2: “gather*” OR “crowd*”<br>#3: “influenza” OR “flu”<br>#4: #1 AND #2 AND #3	October 18, 2018	H.G., E.S.

Findings

We identified 3 studies for the systematic review after reviewing 815 titles and 121 abstracts identified from the 4 databases and other sources. Appendix Figure 9 shows the study selection process. Among these 3 articles, 2 were based on the 1918 influenza pandemic, and 1 focused on an influenza outbreak during the World Youth Day gathering in 2008 (details shown in Appendix Table 23).

Hachett et al. (*57*) and Markel et al. (*5*). reported a strong association between the early implementation of interventions and the mitigation of the 1918 pandemic. The study by Markel et al. (*5*) showed 3 major categories for NPI: SC, cancellation of public gatherings, and isolation or quarantine in 43 cities in the United States. SC combined with a ban on public gatherings was the most common intervention with a median duration of 4 weeks, which reduced significantly weekly excess death rate Early implementation led to greater delays in reaching peak mortality

rates (Spearman ρ = –0.74, $p<0.001$), lower peak mortality rates (Spearman ρ = 0.31, p = 0.02) and lower total mortality rates (Spearman ρ = 0.37, p = 0.008) (*5*). There was a significant association between increased duration of interventions and a reduction in the total mortality rate (Spearman ρ = –0.39, p = 0.005) (*5*). Another study by Hatchett et al. also focused on the early bans on public gathering and closure of public places in reducing the excess death rate (*57*). In addition, during the 1-week long World Youth Day event in 2008, the group of youths who were accommodated in a single large place (17.2%) had a significantly higher attack rate compared with youths who lived in small classrooms (9.2%) ($p<0.01$) (*157*).

Appendix Table 23. Summary of studies included in the review of avoiding crowding

Study	Influenza	Intervention	Comparison	Outcome
Hatchett RJ, 2007 (*57*)	1918 Pandemic	Early church closure, theater closure and bans on public gathering	Cities with different timing and combination of non-pharmaceutical interventions	Associated with lower peak excess death rates (Spearman ρ = 0.56, ρ = 0.56, ρ = 0.46 separately)
Markel H, 2007 (*5*)	1918 Pandemic	Multiple: SC + cancellation of public gatherings + isolation and quarantine.	Cities with different timing, duration and combination of non-pharmaceutical interventions	Implemented earlier and longer duration are significantly associated with the reduction of influenza transmission
Staff M, 2011 (*157*)	World Youth Day 2008 pilgrims	Pilgrims was sub-divided into smaller groups and accommodated in classrooms for 1 week.	Pilgrims was accommodated as a large group in a gymnasium	The attack rate was significantly ($p<0.01$) higher among pilgrims accommodated in the gymnasium (17.2%) than those staying in the classrooms (9.2%)

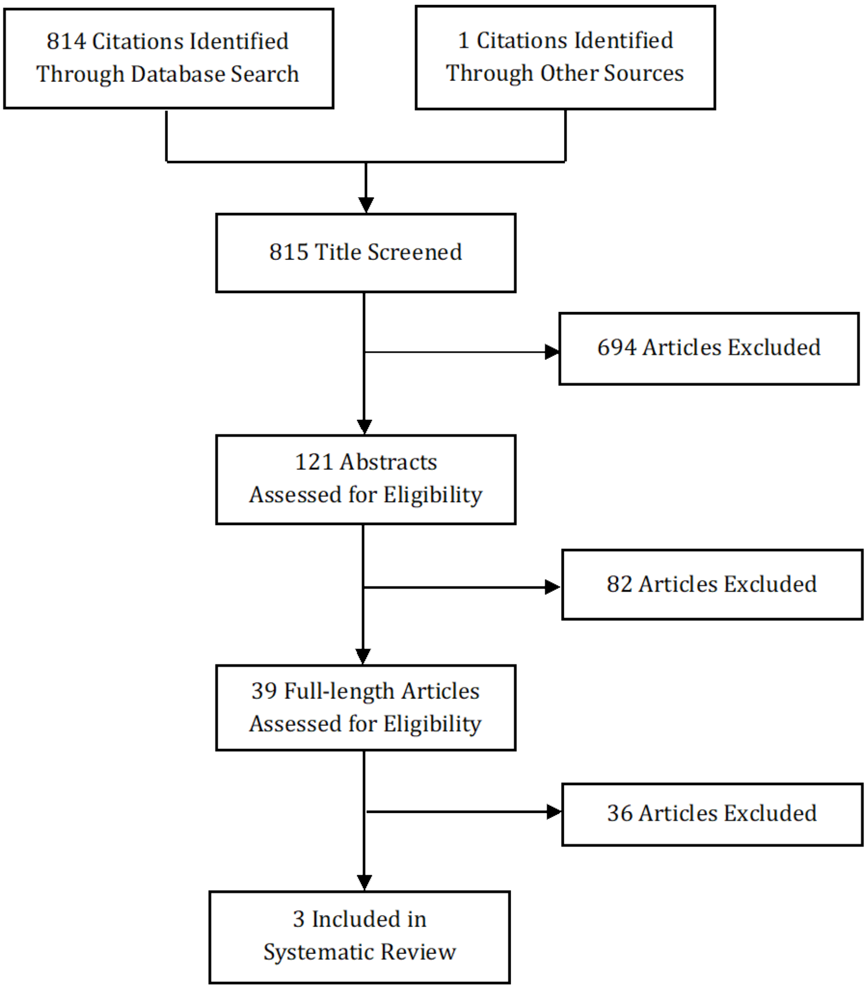

Appendix Figure 9. Flowchart of literature search and study selection for avoiding crowding.

References

1. European Centre for Disease Prevention and Control. Guide to public health measures to reduce the impact of influenza pandemics in Europe: The ECDC Menu. Stockholm: The Centre; 2009.

2. Centers for Disease Control and Prevention. Community mitigation guidelines to prevent pandemic influenza—United States, 2017 [cited 2020 Jan 31]. https://www.cdc.gov/mmwr/volumes/66/rr/rr6601a1.htm

3. Chu CY, de Silva UC, Guo JP, Wang Y, Wen L, Lee VJ, et al. Combined interventions for mitigation of an influenza A (H1N1) 2009 outbreak in a physical training camp in Beijing, China. Int J Infect Dis. 2017;60:77–82. PubMed https://doi.org/10.1016/j.ijid.2017.04.021

4. Gaillat J, Dennetière G, Raffin-Bru E, Valette M, Blanc MC. Summer influenza outbreak in a home for the elderly: application of preventive measures. J Hosp Infect. 2008;70:272–7. PubMed https://doi.org/10.1016/j.jhin.2008.07.009

5. Markel H, Lipman HB, Navarro JA, Sloan A, Michalsen JR, Stern AM, et al. Nonpharmaceutical interventions implemented by US cities during the 1918–1919 influenza pandemic. JAMA. 2007;298:644–54. PubMed https://doi.org/10.1001/jama.298.6.644

6. Vera DM, Hora RA, Murillo A, Wong JF, Torre AJ, Wang D, et al. Assessing the impact of public health interventions on the transmission of pandemic H1N1 influenza a virus aboard a Peruvian navy ship. Influenza Other Respir Viruses. 2014;8:353–9. PubMed https://doi.org/10.1111/irv.12240

7. Ferguson NM, Cummings DA, Fraser C, Cajka JC, Cooley PC, Burke DS. Strategies for mitigating an influenza pandemic. Nature. 2006;442:448–52. PubMed https://doi.org/10.1038/nature04795

8. Flahault A, Vergu E, Coudeville L, Grais RF. Strategies for containing a global influenza pandemic. Vaccine. 2006;24:6751–5. PubMed https://doi.org/10.1016/j.vaccine.2006.05.079

9. Fraser C, Riley S, Anderson RM, Ferguson NM. Factors that make an infectious disease outbreak controllable. Proc Natl Acad Sci U S A. 2004;101:6146–51. PubMed https://doi.org/10.1073/pnas.0307506101

10. Halloran ME, Ferguson NM, Eubank S, Longini IM Jr, Cummings DA, Lewis B, et al. Modeling targeted layered containment of an influenza pandemic in the United States. Proc Natl Acad Sci U S A. 2008;105:4639–44. PubMed https://doi.org/10.1073/pnas.0706849105

11. Kelso JK, Milne GJ, Kelly H. Simulation suggests that rapid activation of social distancing can arrest epidemic development due to a novel strain of influenza. BMC Public Health. 2009;9:117. PubMed https://doi.org/10.1186/1471-2458-9-117

12. Saunders-Hastings P, Quinn Hayes B, Smith R, Krewski D. Modelling community-control strategies to protect hospital resources during an influenza pandemic in Ottawa, Canada. PLoS One. 2017;12:e0179315. PubMed https://doi.org/10.1371/journal.pone.0179315

13. Wang L, Zhang Y, Huang T, Li X. Estimating the value of containment strategies in delaying the arrival time of an influenza pandemic: a case study of travel restriction and patient isolation. Phys Rev E Stat Nonlin Soft Matter Phys. 2012;86:032901. PubMed https://doi.org/10.1103/PhysRevE.86.032901

14. Wu JT, Riley S, Fraser C, Leung GM. Reducing the impact of the next influenza pandemic using household-based public health interventions. PLoS Med. 2006;3:e361. PubMed https://doi.org/10.1371/journal.pmed.0030361

15. Zhang Q, Wang D. Antiviral prophylaxis and isolation for the control of pandemic influenza. Int J Environ Res Public Health. 2014;11:7690–712. PubMed https://doi.org/10.3390/ijerph110807690

16. Zhang Q, Wang D. Assessing the role of voluntary self-isolation in the control of pandemic influenza using a household epidemic model. Int J Environ Res Public Health. 2015;12:9750–67. PubMed https://doi.org/10.3390/ijerph120809750

17. Yasuda H, Suzuki K. Measures against transmission of pandemic H1N1 influenza in Japan in 2009: simulation model. Euro Surveill. 2009;14:19385. PubMed

18. Bell D, Nicoll A, Fukuda K, Horby P, Monto A, Hayden F, et al.; World Health Organization Writing Group. Non-pharmaceutical interventions for pandemic influenza, international measures. Emerg Infect Dis. 2006;12:81–7. PubMed https://doi.org/10.3201/eid1201.051370

19. Peak CM, Childs LM, Grad YH, Buckee CO. Comparing nonpharmaceutical interventions for containing emerging epidemics. Proc Natl Acad Sci U S A. 2017;114:4023–8. PubMed https://doi.org/10.1073/pnas.1616438114

20. An der Heiden M, Buchholz U, Krause G, Kirchner G, Claus H, Haas WH. Breaking the waves: modelling the potential impact of public health measures to defer the epidemic peak of novel influenza A/H1N1. PLoS One. 2009;4:e8356. PubMed https://doi.org/10.1371/journal.pone.0008356

21. McLeod MA, Baker M, Wilson N, Kelly H, Kiedrzynski T, Kool JL. Protective effect of maritime quarantine in South Pacific jurisdictions, 1918–19 influenza pandemic. Emerg Infect Dis. 2008;14:468–70. PubMed https://doi.org/10.3201/eid1403.070927

22. Fujita M, Sato H, Kaku K, Tokuno S, Kanatani Y, Suzuki S, et al. Airport quarantine inspection, follow-up observation, and the prevention of pandemic influenza. Aviat Space Environ Med. 2011;82:782–9. PubMed https://doi.org/10.3357/ASEM.3023.2011

23. Longini IM Jr, Nizam A, Xu S, Ungchusak K, Hanshaoworakul W, Cummings DA, et al. Containing pandemic influenza at the source. Science. 2005;309:1083–7. PubMed https://doi.org/10.1126/science.1115717

24. Nishiura H, Wilson N, Baker MG. Quarantine for pandemic influenza control at the borders of small island nations. BMC Infect Dis. 2009;9:27. PubMed https://doi.org/10.1186/1471-2334-9-27

25. Roberts MG, Baker M, Jennings LC, Sertsou G, Wilson N. A model for the spread and control of pandemic influenza in an isolated geographical region. J R Soc Interface. 2007;4:325–30. PubMed https://doi.org/10.1098/rsif.2006.0176

26. Sato H, Nakada H, Yamaguchi R, Imoto S, Miyano S, Kami M. When should we intervene to control the 2009 influenza A(H1N1) pandemic? Euro Surveill. 2010;15:19455. PubMed https://doi.org/10.2807/ese.15.01.19455-en

27. Yang Y, Atkinson PM, Ettema D. Analysis of CDC social control measures using an agent-based simulation of an influenza epidemic in a city. BMC Infect Dis. 2011;11:199. PubMed https://doi.org/10.1186/1471-2334-11-199

28. Li X, Geng W, Tian H, Lai D. Was mandatory quarantine necessary in China for controlling the 2009 H1N1 pandemic? Int J Environ Res Public Health. 2013;10:4690–700. PubMed https://doi.org/10.3390/ijerph10104690

29. Miyaki K, Sakurazawa H, Mikurube H, Nishizaka M, Ando H, Song Y, et al. An effective quarantine measure reduced the total incidence of influenza A H1N1 in the workplace: another way to control the H1N1 flu pandemic. J Occup Health. 2011;53:287–92. PubMed https://doi.org/10.1539/joh.10-0024-FS

30. van Gemert C, Hellard M, McBryde ES, Fielding J, Spelman T, Higgins N, et al. Intrahousehold transmission of pandemic (H1N1) 2009 virus, Victoria, Australia. Emerg Infect Dis. 2011;17:1599–607. PubMed https://doi.org/10.3201/eid1709.101948

31. Cauchemez S, Ferguson NM, Wachtel C, Tegnell A, Saour G, Duncan B, et al. Closure of schools during an influenza pandemic. Lancet Infect Dis. 2009;9:473–81. PubMed https://doi.org/10.1016/S1473-3099(09)70176-8

32. Jackson C, Vynnycky E, Hawker J, Olowokure B, Mangtani P. School closures and influenza: systematic review of epidemiological studies. BMJ Open. 2013;3:e002149. PubMed https://doi.org/10.1136/bmjopen-2012-002149

33. Cauchemez S, Bhattarai A, Marchbanks TL, Fagan RP, Ostroff S, Ferguson NM, et al.; Pennsylvania H1N1 working group. Role of social networks in shaping disease transmission during a community outbreak of 2009 H1N1 pandemic influenza. Proc Natl Acad Sci U S A. 2011;108:2825–30. PubMed https://doi.org/10.1073/pnas.1008895108

34. Chen T, Huang Y, Liu R, Xie Z, Chen S, Hu G. Evaluating the effects of common control measures for influenza A (H1N1) outbreak at school in China: a modeling study. PLoS One. 2017;12:e0177672. PubMed https://doi.org/10.1371/journal.pone.0177672

35. Chen T, Zhao B, Liu R, Zhang X, Xie Z, Chen S. Simulation of key interventions for seasonal influenza outbreak control at school in Changsha, China. J Int Med Res. 2018:300060518764268. PubMed https://doi.org/10.1177/0300060518764268

36. Davis BM, Markel H, Navarro A, Wells E, Monto AS, Aiello AE. The effect of reactive school closure on community influenza-like illness counts in the state of Michigan during the 2009 H1N1 pandemic. Clin Infect Dis. 2015;60:e90–7. PubMed https://doi.org/10.1093/cid/civ182

37. Egger JR, Konty KJ, Wilson E, Karpati A, Matte T, Weiss D, et al. The effect of school dismissal on rates of influenza-like illness in New York City schools during the spring 2009 novel H1N1 outbreak. J Sch Health. 2012;82:123–30. PubMed https://doi.org/10.1111/j.1746-1561.2011.00675.x

38. Hens N, Calatayud L, Kurkela S, Tamme T, Wallinga J. Robust reconstruction and analysis of outbreak data: influenza A(H1N1)v transmission in a school-based population. Am J Epidemiol. 2012;176:196–203. PubMed https://doi.org/10.1093/aje/kws006

39. Janjua NZ, Skowronski DM, Hottes TS, Osei W, Adams E, Petric M, et al. Seasonal influenza vaccine and increased risk of pandemic A/H1N1-related illness: first detection of the association in British Columbia, Canada. Clin Infect Dis. 2010;51:1017–27. PubMed https://doi.org/10.1086/656586

40. Kawano S, Kakehashi M. Substantial impact of school closure on the transmission dynamics during the pandemic flu H1N1–2009 in Oita, Japan. PLoS One. 2015;10:e0144839. PubMed https://doi.org/10.1371/journal.pone.0144839

41. Loustalot F, Silk BJ, Gaither A, Shim T, Lamias M, Dawood F, et al. Household transmission of 2009 pandemic influenza A (H1N1) and nonpharmaceutical interventions among households of high school students in San Antonio, Texas. Clin Infect Dis. 2011;52(Suppl 1):S146–53. PubMed https://doi.org/10.1093/cid/ciq057

42. Miller JC, Danon L, O'Hagan JJ, Goldstein E, Lajous M, Lipsitch M. Student behavior during a school closure caused by pandemic influenza A/H1N1. PLoS One. 2010;5:e10425. PubMed https://doi.org/10.1371/journal.pone.0010425

43. Russell ES, Zheteyeva Y, Gao H, Shi J, Rainey JJ, Thoroughman D, et al. Reactive school closure during increased influenza-like illness (ILI) activity in western Kentucky, 2013: a field evaluation of effect on ILI incidence and economic and social consequences for families. Open Forum Infect Dis. 2016;3:ofw113. PubMed https://doi.org/10.1093/ofid/ofw113

44. Sato T, Akita T, Tanaka J. Evaluation of strategies for control and prevention of pandemic influenza (H1N1pdm) in Japanese children attending school in a rural town. Simulation using mathematical models. Nihon Koshu Eisei Zasshi. 2013;60:204–11. PubMed

45. Sonoguchi T, Naito H, Hara M, Takeuchi Y, Fukumi H. Cross-subtype protection in humans during sequential, overlapping, and/or concurrent epidemics caused by H3N2 and H1N1 influenza viruses. J Infect Dis. 1985;151:81–8. PubMed https://doi.org/10.1093/infdis/151.1.81

46. Sugisaki K, Seki N, Tanabe N, Saito R, Sasaki A, Sasaki S, et al. Effective school actions for mitigating seasonal influenza outbreaks in Niigata, Japan. PLoS One. 2013;8:e74716. PubMed https://doi.org/10.1371/journal.pone.0074716

47. Uchida M, Tsukahara T, Kaneko M, Washizuka S, Kawa S. Swine-origin influenza A outbreak 2009 at Shinshu University, Japan. BMC Public Health. 2011;11:79. PubMed https://doi.org/10.1186/1471-2458-11-79

48. Uchida M, Tsukahara T, Kaneko M, Washizuka S, Kawa S. Effect of short-term school closures on the H1N1 pandemic in Japan: a comparative case study. Infection. 2012;40:549–56. PubMed https://doi.org/10.1007/s15010-012-0304-z

49. Bolton KJ, McCaw JM, Moss R, Morris RS, Wang S, Burma A, et al. Likely effectiveness of pharmaceutical and non-pharmaceutical interventions for mitigating influenza virus transmission in Mongolia. Bull World Health Organ. 2012;90:264–71. PubMed https://doi.org/10.2471/BLT.11.093419

50. Bootsma MC, Ferguson NM. The effect of public health measures on the 1918 influenza pandemic in U.S. cities. Proc Natl Acad Sci U S A. 2007;104:7588–93. PubMed https://doi.org/10.1073/pnas.0611071104

51. Caley P, Philp DJ, McCracken K. Quantifying social distancing arising from pandemic influenza. J R Soc Interface. 2008;5:631–9. PubMed https://doi.org/10.1098/rsif.2007.1197

52. Chowell G, Echevarría-Zuno S, Viboud C, Simonsen L, Tamerius J, Miller MA, et al. Characterizing the epidemiology of the 2009 influenza A/H1N1 pandemic in Mexico. PLoS Med. 2011;8:e1000436. PubMed https://doi.org/10.1371/journal.pmed.1000436

53. Copeland DL, Basurto-Davila R, Chung W, Kurian A, Fishbein DB, Szymanowski P, et al. Effectiveness of a school district closure for pandemic influenza A (H1N1) on acute respiratory illnesses in the community: a natural experiment. Clin Infect Dis. 2013;56:509–16. PubMed https://doi.org/10.1093/cid/cis890

54. Cowling BJ, Lau EH, Lam CL, Cheng CK, Kovar J, Chan KH, et al. Effects of school closures, 2008 winter influenza season, Hong Kong. Emerg Infect Dis. 2008;14:1660–2. PubMed https://doi.org/10.3201/eid1410.080646

55. Cruz-Pacheco G, Duran L, Esteva L, Minzoni A, Lopez-Cervantes M, Panayotaros P, et al. Modelling of the influenza A(H1N1)v outbreak in Mexico City, April–May 2009, with control sanitary measures. Euro Surveill. 2009;14:19254. PubMed

56. Cowling BJ, Lau MS, Ho LM, Chuang SK, Tsang T, Liu SH, et al. The effective reproduction number of pandemic influenza: prospective estimation. Epidemiology. 2010;21:842–6. PubMed https://doi.org/10.1097/EDE.0b013e3181f20977

57. Hatchett RJ, Mecher CE, Lipsitch M. Public health interventions and epidemic intensity during the 1918 influenza pandemic. Proc Natl Acad Sci U S A. 2007;104:7582–7. PubMed https://doi.org/10.1073/pnas.0610941104

58. Herrera-Valdez MA, Cruz-Aponte M, Castillo-Chavez C. Multiple outbreaks for the same pandemic: Local transportation and social distancing explain the different “waves” of A-H1N1pdm cases observed in México during 2009. Math Biosci Eng. 2011;8:21–48. PubMed https://doi.org/10.3934/mbe.2011.8.21

59. Tinoco Y, Razuri H, Ortiz EJ, Gomez J, Widdowson MA, Uyeki T, et al.; Peru Influenza Working Group. Preliminary population-based epidemiological and clinical data on 2009 pandemic H1N1 influenza A (pH1N1) from Lima, Peru. Influenza Other Respir Viruses. 2009;3:253–6. PubMed https://doi.org/10.1111/j.1750-2659.2009.00111.x

60. Wu JT, Cowling BJ, Lau EH, Ip DK, Ho LM, Tsang T, et al. School closure and mitigation of pandemic (H1N1) 2009, Hong Kong. Emerg Infect Dis. 2010;16:538–41. PubMed https://doi.org/10.3201/eid1603.091216

61. Ali ST, Kadi AS, Ferguson NM. Transmission dynamics of the 2009 influenza A (H1N1) pandemic in India: the impact of holiday-related school closure. Epidemics. 2013;5:157–63. PubMed https://doi.org/10.1016/j.epidem.2013.08.001

62. Baguelin M, Hoek AJ, Jit M, Flasche S, White PJ, Edmunds WJ. Vaccination against pandemic influenza A/H1N1v in England: a real-time economic evaluation. Vaccine. 2010;28:2370–84. PubMed https://doi.org/10.1016/j.vaccine.2010.01.002

63. Birrell PJ, Ketsetzis G, Gay NJ, Cooper BS, Presanis AM, Harris RJ, et al. Bayesian modeling to unmask and predict influenza A/H1N1pdm dynamics in London. Proc Natl Acad Sci U S A. 2011;108:18238–43. PubMed https://doi.org/10.1073/pnas.1103002108

64. Cauchemez S, Valleron AJ, Boëlle PY, Flahault A, Ferguson NM. Estimating the impact of school closure on influenza transmission from Sentinel data. Nature. 2008;452:750–4. PubMed https://doi.org/10.1038/nature06732

65. Chowell G, Towers S, Viboud C, Fuentes R, Sotomayor V. Rates of influenza-like illness and winter school breaks, Chile, 2004–2010. Emerg Infect Dis. 2014;20:1203–7. PubMed https://doi.org/10.3201/eid2007.130967

66. Chowell G, Viboud C, Munayco CV, Gómez J, Simonsen L, Miller MA, et al. Spatial and temporal characteristics of the 2009 A/H1N1 influenza pandemic in Peru. PLoS One. 2011;6:e21287. PubMed https://doi.org/10.1371/journal.pone.0021287

67. Chu Y, Wu Z, Ji J, Sun J, Sun X, Qin G, et al. Effects of school breaks on influenza-like illness incidence in a temperate Chinese region: an ecological study from 2008 to 2015. BMJ Open. 2017;7:e013159. PubMed https://doi.org/10.1136/bmjopen-2016-013159

68. Davies JR, Grilli EA, Smith AJ, Hoskins TW. Prophylactic use of amantadine in a boarding school outbreak of influenza A. J R Coll Gen Pract. 1988;38:346–8. PubMed

69. Eames KT, Tilston NL, Brooks-Pollock E, Edmunds WJ. Measured dynamic social contact patterns explain the spread of H1N1v influenza. PLOS Comput Biol. 2012;8:e1002425. PubMed https://doi.org/10.1371/journal.pcbi.1002425

70. Earn DJ, He D, Loeb MB, Fonseca K, Lee BE, Dushoff J. Effects of school closure on incidence of pandemic influenza in Alberta, Canada. Ann Intern Med. 2012;156:173–81. PubMed https://doi.org/10.7326/0003-4819-156-3-201202070-00005

71. Evans B, Charlett A, Powers C, McLean E, Zhao H, Bermingham A, et al. Has estimation of numbers of cases of pandemic influenza H1N1 in England in 2009 provided a useful measure of the occurrence of disease? Influenza Other Respir Viruses. 2011;5:e504–12. PubMed https://doi.org/10.1111/j.1750-2659.2011.00259.x

72. Ewing A, Lee EC, Viboud C, Bansal S. Contact, travel, and transmission: the impact of winter holidays on influenza dynamics in the United States. J Infect Dis. 2017;215:732–9. PubMed

73. Flasche S, Hens N, Boëlle PY, Mossong J, van Ballegooijen WM, Nunes B, et al. Different transmission patterns in the early stages of the influenza A(H1N1)v pandemic: a comparative analysis of 12 European countries. Epidemics. 2011;3:125–33. PubMed https://doi.org/10.1016/j.epidem.2011.03.005

74. Fujii H, Takahashi H, Ohyama T, Hattori K, Suzuki S. Evaluation of the school health surveillance system for influenza, Tokyo, 1999–2000. Jpn J Infect Dis. 2002;55:97–9. PubMed

75. Garza RC, Basurto-Dávila R, Ortega-Sanchez IR, Carlino LO, Meltzer MI, Albalak R, et al. Effect of winter school breaks on influenza-like illness, Argentina, 2005–2008. Emerg Infect Dis. 2013;19:938–44. PubMed https://doi.org/10.3201/eid1906.120916

76. Louie JK, Schnurr DP, Guevara HF, Honarmand S, Cheung M, Cottam D, et al. Creating a model program for influenza surveillance in California: results from the 2005–2006 influenza season. Am J Prev Med. 2007;33:353–7. PubMed https://doi.org/10.1016/j.amepre.2007.05.008

77. Luca G, Kerckhove KV, Coletti P, Poletto C, Bossuyt N, Hens N, et al. The impact of regular school closure on seasonal influenza epidemics: a data-driven spatial transmission model for Belgium. BMC Infect Dis. 2018;18:29. PubMed https://doi.org/10.1186/s12879-017-2934-3

78. Merler S, Ajelli M, Pugliese A, Ferguson NM. Determinants of the spatiotemporal dynamics of the 2009 H1N1 pandemic in Europe: implications for real-time modelling. PLOS Comput Biol. 2011;7:e1002205. PubMed https://doi.org/10.1371/journal.pcbi.1002205

79. Monto AS, Davenport FM, Napier JA, Francis T Jr. Modification of an outbreak of influenza in Tecumseh, Michigan by vaccination of schoolchildren. J Infect Dis. 1970;122:16–25. PubMed https://doi.org/10.1093/infdis/122.1-2.16

80. Rodriguez CV, Rietberg K, Baer A, Kwan-Gett T, Duchin J. Association between school closure and subsequent absenteeism during a seasonal influenza epidemic. Epidemiology. 2009;20:787–92. PubMed https://doi.org/10.1097/EDE.0b013e3181b5f3ec

81. Smith S, Smith GE, Olowokure B, Ibbotson S, Foord D, Maguire H, et al. Early spread of the 2009 influenza A(H1N1) pandemic in the United Kingdom—use of local syndromic data, May-August 2009. Euro Surveill. 2011;16:19771. PubMed

82. Te Beest DE, Birrell PJ, Wallinga J, De Angelis D, van Boven M. Joint modelling of serological and hospitalization data reveals that high levels of pre-existing immunity and school holidays shaped the influenza A pandemic of 2009 in the Netherlands. J R Soc Interface. 2015;12:20141244. PubMed https://doi.org/10.1098/rsif.2014.1244

83. Wheeler CC, Erhart LM, Jehn ML. Effect of school closure on the incidence of influenza among school-age children in Arizona. Public Health Rep. 2010;125:851–9. PubMed https://doi.org/10.1177/003335491012500612

84. Wu J, Xu F, Lu L, Lu M, Miao L, Gao T, et al. Safety and effectiveness of a 2009 H1N1 vaccine in Beijing. N Engl J Med. 2010;363:2416–23. PubMed https://doi.org/10.1056/NEJMoa1006736

85. Yu H, Cauchemez S, Donnelly CA, Zhou L, Feng L, Xiang N, et al. Transmission dynamics, border entry screening, and school holidays during the 2009 influenza A (H1N1) pandemic, China. Emerg Infect Dis. 2012;18:758–66. PubMed https://doi.org/10.3201/eid1805.110356

86. Armstrong C, Hopkins R. An epidemiological study of the 1920 epidemic of influenza in an isolated rural community. Public Health Rep. 1921;36:1671–702. https://doi.org/10.2307/4576063

87. Baker MG, Wilson N, Huang QS, Paine S, Lopez L, Bandaranayake D, et al. Pandemic influenza A(H1N1)v in New Zealand: the experience from April to August 2009. Euro Surveill. 2009;14:19319. PubMed https://doi.org/10.2807/ese.14.34.19319-en

88. Briscoe JH. The protective effect of influenza vaccine in a mixed influenza A and B epidemic in a boys' boarding school. J R Coll Gen Pract. 1977;27:28–31. PubMed

89. Calatayud L, Kurkela S, Neave PE, Brock A, Perkins S, Zuckerman M, et al. Pandemic (H1N1) 2009 virus outbreak in a school in London, April-May 2009: an observational study. Epidemiol Infect. 2010;138:183–91. PubMed https://doi.org/10.1017/S0950268809991191

90. Carrillo-Santisteve P, Renard-Dubois S, Cheron G, Csaszar-Goutchkoff M, Lecuit M, Lortholary O, et al. 2009 pandemic influenza A(H1N1) outbreak in a complex of schools in Paris, France, June 2009. Euro Surveill. 2010;15:19599. PubMed https://doi.org/10.2807/ese.15.25.19599-en

91. Cashman P, Massey P, Durrheim D, Islam F, Merritt T, Eastwood K. Pneumonia cluster in a boarding school—implications for influenza control. Commun Dis Intell Q Rep. 2007;31:296–8. PubMed

92. Chieochansin T, Makkoch J, Suwannakarn K, Payungporn S, Poovorawan Y. Novel H1N1 2009 influenza virus infection in Bangkok, Thailand: effects of school closures. Asian Biomed. 2009;3:469–75.

93. Cohen NJ, Callahan DB, Gonzalez V, Balaban V, Wang RT, Pordell P, et al. Respiratory illness in households of school-dismissed students during pandemic (H1N1) 2009. Emerg Infect Dis. 2011;17:1756–7. PubMed https://doi.org/10.3201/eid1709.101589

94. Danis K, Fitzgerald M, Connell J, Conlon M, Murphy PG. Lessons from a pre-season influenza outbreak in a day school. Commun Dis Public Health. 2004;7:179–83. PubMed

95. Echevarría-Zuno S, Mejía-Aranguré JM, Mar-Obeso AJ, Grajales-Muñiz C, Robles-Pérez E, González-León M, et al. Infection and death from influenza A H1N1 virus in Mexico: a retrospective analysis. Lancet. 2009;374:2072–9. PubMed https://doi.org/10.1016/S0140-6736(09)61638-X

96. Effler PV, Carcione D, Giele C, Dowse GK, Goggin L, Mak DB. Household responses to pandemic (H1N1) 2009-related school closures, Perth, Western Australia. Emerg Infect Dis. 2010;16:205–11. PubMed https://doi.org/10.3201/eid1602.091372

97. Engelhard D, Bromberg M, Averbuch D, Tenenbaum A, Goldmann D, Kunin M, et al. Increased extent of and risk factors for pandemic (H1N1) 2009 and seasonal influenza among children, Israel. Emerg Infect Dis. 2011;17:1740–3. PubMed https://doi.org/10.3201/eid1709.102022

98. Farley TA, St Germain JM, Chamberlain LA, Krassner L. The impact of influenza vaccination on respiratory illness at a boarding school. J Am Coll Health. 1992;41:127–31. PubMed https://doi.org/10.1080/07448481.1992.9936312

99. Glass RI, Brann EA, Slade JD, Jones WE, Scally MJ, Craven RB, et al. Community-wide surveillance of influenza after outbreaks due to H3N2 (A/Victoria/75 and A/Texas/77) and H1N1 (A/USSR/77) influenza viruses, Mercer County, New Jersey, 1978. J Infect Dis. 1978;138:703–6. PubMed https://doi.org/10.1093/infdis/138.5.703

100. Gomez J, Munayco C, Arrasco J, Suarez L, Laguna-Torres V, Aguilar P, et al. Pandemic influenza in a southern hemisphere setting: the experience in Peru from May to September, 2009. Euro Surveill. 2009;14:19371. PubMed https://doi.org/10.2807/ese.14.42.19371-en

101. Grilli EA, Anderson MJ, Hoskins TW. Concurrent outbreaks of influenza and parvovirus B19 in a boys' boarding school. Epidemiol Infect. 1989;103:359–69. PubMed https://doi.org/10.1017/S0950268800030697

102. Guinard A, Grout L, Durand C, Schwoebel V. Outbreak of influenza A(H1N1)v without travel history in a school in the Toulouse district, France, June 2009. Euro Surveill. 2009;14:19265. PubMed https://doi.org/10.2807/ese.14.27.19265-en

103. Health Protection Agency West Midlands H1N1v Investigation Team. Preliminary descriptive epidemiology of a large school outbreak of influenza A(H1N1)v in the West Midlands, United Kingdom, May 2009. Euro Surveill. 2009;14:19264. PubMed

104. Heymann A, Chodick G, Reichman B, Kokia E, Laufer J. Influence of school closure on the incidence of viral respiratory diseases among children and on health care utilization. Pediatr Infect Dis J. 2004;23:675–7. PubMed https://doi.org/10.1097/01.inf.0000128778.54105.06

105. Heymann AD, Hoch I, Valinsky L, Kokia E, Steinberg DM. School closure may be effective in reducing transmission of respiratory viruses in the community. Epidemiol Infect. 2009;137:1369–76. PubMed https://doi.org/10.1017/S0950268809002556

106. Hsueh PR, Lee PI, Chiu AWH, Yen MY. Pandemic (H1N1) 2009 vaccination and class suspensions after outbreaks, Taipei City, Taiwan. Emerg Infect Dis. 2010;16:1309–11. PubMed https://doi.org/10.3201/eid1608.100310

107. Huai Y, Lin J, Varma JK, Peng Z, He J, Cheng C, et al. A primary school outbreak of pandemic 2009 influenza A (H1N1) in China. Influenza Other Respir Viruses. 2010;4:259–66. PubMed https://doi.org/10.1111/j.1750-2659.2010.00150.x

108. Janusz KB, Cortes JE, Serdarevic F, Jones RC, Jones JD, Ritger KA, et al.; Chicago H1N1 Epidemiology Team. Influenza-like illness in a community surrounding a school-based outbreak of 2009 pandemic influenza A (H1N1) virus: Chicago, Illinois, 2009. Clin Infect Dis. 2011;52(Suppl 1):S94–101. PubMed https://doi.org/10.1093/cid/ciq025

109. Johnson AJ, Moore ZS, Edelson PJ, Kinnane L, Davies M, Shay DK, et al. Household responses to school closure resulting from outbreak of influenza B, North Carolina. Emerg Infect Dis. 2008;14:1024–30. PubMed https://doi.org/10.3201/eid1407.080096

110. Jordan EO, Reed DB, Fink EB. Influenza in three Chicago groups. J Infect Dis. 1919;25:74–95. https://doi.org/10.1093/infdis/25.1.74

111. Kawaguchi R, Miyazono M, Noda T, Takayama Y, Sasai Y, Iso H. Influenza (H1N1) 2009 outbreak and school closure, Osaka Prefecture, Japan. Emerg Infect Dis. 2009;15:1685. PubMed https://doi.org/10.3201/eid1510.091029

112. Lajous M, Danon L, López-Ridaura R, Astley CM, Miller JC, Dowell SF, et al. Mobile messaging as surveillance tool during pandemic (H1N1) 2009, Mexico. Emerg Infect Dis. 2010;16:1488–9. PubMed https://doi.org/10.3201/eid1609.100671

113. Leonida DD. Morbidity patterns reflected in a school health program during an influenza epidemic season. IMJ Ill Med J. 1970;137:262–4. PubMed

114. Lessler J, Reich NG, Cummings DA, Nair HP, Jordan HT, Thompson N; New York City Department of Health and Mental Hygiene Swine Influenza Investigation Team. Outbreak of 2009 pandemic influenza A (H1N1) at a New York City school. N Engl J Med. 2009;361:2628–36. PubMed https://doi.org/10.1056/NEJMoa0906089

115. Leung YH, Li MP, Chuang SK. A school outbreak of pandemic (H1N1) 2009 infection: assessment of secondary household transmission and the protective role of oseltamivir. Epidemiol Infect. 2011;139:41–4. PubMed https://doi.org/10.1017/S0950268810001445

116. Marchbanks TL, Bhattarai A, Fagan RP, Ostroff S, Sodha SV, Moll ME, et al.; Pennsylvania Working Group. An outbreak of 2009 pandemic influenza A (H1N1) virus infection in an elementary school in Pennsylvania. Clin Infect Dis. 2011;52(Suppl 1):S154–60. PubMed https://doi.org/10.1093/cid/ciq058

117. Miller DL, Lee JA. Influenza in Britain 1967–68. J Hyg (Lond). 1969;67:559–72. PubMed https://doi.org/10.1017/S0022172400042005

118. Nishiura H, Castillo-Chavez C, Safan M, Chowell G. Transmission potential of the new influenza A(H1N1) virus and its age-specificity in Japan. Euro Surveill. 2009;14:19227. PubMed https://doi.org/10.2807/ese.14.22.19227-en

119. Olson JG. School absenteeism during an outbreak of B/Hong Kong/5/72-like influenza virus in Taipei, Taiwan. Southeast Asian J Trop Med Public Health. 1980;11:429–34. PubMed

120. Paine S, Mercer GN, Kelly PM, Bandaranayake D, Baker MG, Huang QS, et al. Transmissibility of 2009 pandemic influenza A(H1N1) in New Zealand: effective reproduction number and influence of age, ethnicity and importations. Euro Surveill. 2010;15:19591. PubMed

121. Petrović V, Seguljev Z, Cosić G, Ristić M, Nedeljković J, Dragnić N, et al. Overview of the winter wave of 2009 pandemic influenza A(H1N1)v in Vojvodina, Serbia. Croat Med J. 2011;52:141–50. PubMed https://doi.org/10.3325/cmj.2011.52.141

122. Poggensee G, Gilsdorf A, Buda S, Eckmanns T, Claus H, Altmann D, et al.; RKI Working Group Pandemic Influenza. The first wave of pandemic influenza (H1N1) 2009 in Germany: from initiation to acceleration. BMC Infect Dis. 2010;10:155. PubMed https://doi.org/10.1186/1471-2334-10-155

123. Rajatonirina S, Heraud JM, Randrianasolo L, Razanajatovo N, Ramandimbisoa T, Ratsitorahina M, et al. Pandemic influenza A(H1N1) 2009 virus outbreak among boarding school pupils in Madagascar: compliance and adverse effects of prophylactic oseltamivir treatment. J Infect Dev Ctries. 2011;5:156–62. PubMed https://doi.org/10.3855/jidc.1318

124. Shaw C, Mclean M, McKenzie J. Other surveillance reports: influenza-like illness in Wellington schools 2005. New Zealand Public Health Surveillance Report. 2006;4:4–6.

125. Shimada T, Gu Y, Kamiya H, Komiya N, Odaira F, Sunagawa T, et al. Epidemiology of influenza A(H1N1)v virus infection in Japan, May–June 2009. Euro Surveill. 2009;14:19244. PubMed https://doi.org/10.2807/ese.14.24.19244-en

126. Lo JY, Tsang TH, Leung Y-H, Yeung EY, Wu T, Lim WW. Respiratory infections during SARS outbreak, Hong Kong, 2003. Emerg Infect Dis. 2005;11:1738–41. PubMed https://doi.org/10.3201/eid1111.050729

127. World Health Organization. Human infection with new influenza A (H1N1) virus: clinical observations from a school-associated outbreak in Kobe, Japan, May 2009. Wkly Epidemiol Rec. 2009;84:237–44. PubMed

128. Smith A, Coles S, Johnson S, Saldana L, Ihekweazu C, O’Moore E. An outbreak of influenza A(H1N1)v in a boarding school in South East England, May–June 2009. Euro Surveill. 2009;14:19263. PubMed https://doi.org/10.2807/ese.14.27.19263-en

129. Strong M, Burrows J, Stedman E, Redgrave P. Adverse drug effects following oseltamivir mass treatment and prophylaxis in a school outbreak of 2009 pandemic influenza A(H1N1) in June 2009, Sheffield, United Kingdom. Euro Surveill. 2010;15:19565. PubMed

130. van Gageldonk-Lafeber AB, Hooiveld M, Meijer A, Donker GA, Veldman-Ariesen MJ, van der Hoek W, et al. The relative clinical impact of 2009 pandemic influenza A (H1N1) in the community compared to seasonal influenza in the Netherlands was most marked among 5–14 year olds. Influenza Other Respir Viruses. 2011;5:e513–20. PubMed https://doi.org/10.1111/j.1750-2659.2011.00260.x

131. Wallensten A, Oliver I, Lewis D, Harrison S. Compliance and side effects of prophylactic oseltamivir treatment in a school in South West England. Euro Surveill. 2009;14:19285. PubMed https://doi.org/10.2807/ese.14.30.19285-en

132. Winslow CE, Rogers JF. Statistics of the 1918 epidemic of influenza in connecticut: with a consideration of the factors which influenced the prevalence of this disease in various communities. J Infect Dis. 1920;26:185–216. https://doi.org/10.1093/infdis/26.3.185

133. Jackson C, Mangtani P, Hawker J, Olowokure B, Vynnycky E. The effects of school closures on influenza outbreaks and pandemics: systematic review of simulation studies. PLoS One. 2014;9:e97297. PubMed https://doi.org/10.1371/journal.pone.0097297

134. Ahmed F, Zviedrite N, Uzicanin A. Effectiveness of workplace social distancing measures in reducing influenza transmission: a systematic review. BMC Public Health. 2018;18:518. PubMed https://doi.org/10.1186/s12889-018-5446-1

135. Kumar S, Quinn SC, Kim KH, Daniel LH, Freimuth VS. The impact of workplace policies and other social factors on self-reported influenza-like illness incidence during the 2009 H1N1 pandemic. Am J Public Health. 2012;102:134–40. PubMed https://doi.org/10.2105/AJPH.2011.300307

136. Lee V, Yap J, Cook AR, Chen M, Tay J, Barr I, et al. Effectiveness of public health measures in mitigating pandemic influenza spread: a prospective sero-epidemiological cohort study. J Infect Dis. 2010;202:1319–26. PubMed https://doi.org/10.1086/656480

137. Rousculp MD, Johnston SS, Palmer LA, Chu BC, Mahadevia PJ, Nichol KL. Attending work while sick: implication of flexible sick leave policies. J Occup Environ Med. 2010;52:1009–13. PubMed https://doi.org/10.1097/JOM.0b013e3181f43844

138. Asfaw A, Rosa R, Pana-Cryan R. Potential economic benefits of paid sick leave in reducing absenteeism related to the spread of influenza-like illness. J Occup Environ Med. 2017;59:822–9. PubMed https://doi.org/10.1097/JOM.0000000000001076

139. Piper K, Youk A, James AE III, Kumar S. Paid sick days and stay-at-home behavior for influenza. PLoS One. 2017;12:e0170698. PubMed https://doi.org/10.1371/journal.pone.0170698

140. Andradóttir S, Chiu W, Goldsman D, Lee ML, Tsui KL, Sander B, et al. Reactive strategies for containing developing outbreaks of pandemic influenza. BMC Public Health. 2011;11(Suppl 1):S1. PubMed https://doi.org/10.1186/1471-2458-11-S1-S1

141. Mao L. Agent-based simulation for weekend-extension strategies to mitigate influenza outbreaks. BMC Public Health. 2011;11:522. PubMed https://doi.org/10.1186/1471-2458-11-522

142. Miller G, Randolph S, Patterson JE. Responding to simulated pandemic influenza in San Antonio, Texas. Infect Control Hosp Epidemiol. 2008;29:320–6. PubMed https://doi.org/10.1086/529212

143. Milne GJ, Baskaran P, Halder N, Karl S, Kelso J. Pandemic influenza in Papua New Guinea: a modelling study comparison with pandemic spread in a developed country. BMJ Open. 2013;3:e002518. PubMed https://doi.org/10.1136/bmjopen-2012-002518

144. Milne GJ, Kelso JK, Kelly HA, Huband ST, McVernon J. A small community model for the transmission of infectious diseases: comparison of school closure as an intervention in individual-based models of an influenza pandemic. PloS One. 2008;3:e4005.

145. Perlroth DJ, Glass RJ, Davey VJ, Cannon D, Garber AM, Owens DK. Health outcomes and costs of community mitigation strategies for an influenza pandemic in the United States. Clin Infect Dis. 2010;50:165–74. PubMed https://doi.org/10.1086/649867

146. Timpka T, Eriksson H, Holm E, Strömgren M, Ekberg J, Spreco A, et al. Relevance of workplace social mixing during influenza pandemics: an experimental modelling study of workplace cultures. Epidemiol Infect. 2016;144:2031–42. PubMed https://doi.org/10.1017/S0950268816000169

147. Xia S, Liu J, Cheung W. Identifying the relative priorities of subpopulations for containing infectious disease spread. PLoS One. 2013;8:e65271. PubMed https://doi.org/10.1371/journal.pone.0065271

148. Zhang T, Fu X, Ma S, Xiao G, Wong L, Kwoh CK, et al. Evaluating temporal factors in combined interventions of workforce shift and school closure for mitigating the spread of influenza. PLoS One. 2012;7:e32203. PubMed https://doi.org/10.1371/journal.pone.0032203

149. Xia H, Nagaraj K, Chen J, Marathe MV. Synthesis of a high resolution social contact network for Delhi with application to pandemic planning. Artif Intell Med. 2015;65:113–30. PubMed https://doi.org/10.1016/j.artmed.2015.06.003

150. Carrat F, Luong J, Lao H, Sallé AV, Lajaunie C, Wackernagel H. A ‘small-world-like’ model for comparing interventions aimed at preventing and controlling influenza pandemics. BMC Med. 2006;4:26. PubMed https://doi.org/10.1186/1741-7015-4-26

151. Mao L. Evaluating the combined effectiveness of influenza control strategies and human preventive behavior. PLoS One. 2011;6:e24706. PubMed https://doi.org/10.1371/journal.pone.0024706

152. Halder N, Kelso JK, Milne GJ. Cost-effective strategies for mitigating a future influenza pandemic with H1N1 2009 characteristics. PLoS One. 2011;6:e22087. PubMed https://doi.org/10.1371/journal.pone.0022087

153. Merler S, Jurman G, Furlanello C, Rizzo C, Bella A, Massari M, et al. Strategies for containing an influenza pandemic: the case of Italy. In: 2006 1st Biol-Inspired Models of Network, Information and Computing Systems. New York: Springer; 2006. p. 1–7.

154. Ciofi degli Atti ML, Merler S, Rizzo C, Ajelli M, Massari M, Manfredi P, et al. Mitigation measures for pandemic influenza in Italy: an individual based model considering different scenarios. PLoS One. 2008;3:e1790. PubMed https://doi.org/10.1371/journal.pone.0001790

155. Rizzo C, Lunelli A, Pugliese A, Bella A, Manfredi P, Tomba GS, et al. Scenarios of diffusion and control of an influenza pandemic in Italy. Epidemiol Infect. 2008;136:1650–7. PubMed https://doi.org/10.1017/S095026880800037X

156. Ferguson NM, Cummings DA, Cauchemez S, Fraser C, Riley S, Meeyai A, et al. Strategies for containing an emerging influenza pandemic in Southeast Asia. Nature. 2005;437:209–14. PubMed https://doi.org/10.1038/nature04017

157. Staff M, Torres MI. An influenza outbreak among pilgrims sleeping at a school without purpose built overnight accommodation facilities. Commun Dis Intell Q Rep. 2011;35:10–5. PubMed
